# Virtual Care Among Adults Facing Language Barriers: A Systematic Review and Meta-Analysis

**DOI:** 10.1001/jamanetworkopen.2025.13906

**Published:** 2025-06-05

**Authors:** Erica Wennberg, Zuhal Mohmand, David D’Arienzo, Safa Majeed Grant, Elizabeth Uleryk, Manav V. Vyas, Susitha Wanigaratne, Astrid Guttmann

**Affiliations:** 1 Institute of Health Policy, Management and Evaluation, Dalla Lana School of Public Health, University of Toronto, Toronto, Ontario, Canada; 2 Temerty Faculty of Medicine, University of Toronto, Toronto, Ontario, Canada; 3 Child Health Evaluative Sciences, SickKids Research Institute, Toronto, Ontario, Canada; 4 Department of Medicine, Queen’s University, Kingston, Ontario, Canada; 5 Montreal Children’s Hospital, Montreal, Quebec, Canada; 6 E.M. Uleryk Consulting, Mississauga, Ontario, Canada; 7 St Michael’s Hospital-Unity Health Toronto, Toronto, Ontario, Canada; 8 ICES, Toronto, Ontario, Canada; 9 Edwin S.H. Leong Centre for Healthy Children, Toronto, Ontario, Canada; 10 Department of Paediatrics, The Hospital for Sick Children, Toronto, Ontario, Canada

## Abstract

**Importance:**

Patients with language barriers may experience difficulties accessing virtual care.

**Objectives:**

To synthesize the literature on the association between language barriers and use of and satisfaction with virtual care among adult patients and among caregivers of pediatric patients in high-income countries.

**Data Sources:**

Four electronic databases, MEDLINE, Embase, PsycINFO, and Web of Science, were searched from inception to March 2023 using a combination of language and virtual care terms.

**Study Selection:**

Eligible studies compared quantitative data on use of or satisfaction with virtual care among adult patients or caregivers of pediatric patients with or without language barriers in high-income countries.

**Data Extraction and Synthesis:**

Data were extracted by 2 independent reviewers using a piloted data extraction form. Risk of bias assessments were performed using the ROBINS-E tool. Data were synthesized by outcome type using random-effects meta-analyses stratified by primary vs specialist care and narrative synthesis. Reporting followed the Preferred Reporting Items for Systematic Reviews and Meta-analyses 2020 guideline.

**Main Outcomes and Measures:**

The primary outcomes were use of virtual care and satisfaction with virtual care among adult patients and among caregivers of pediatric patients with vs without language barriers.

**Results:**

In total, 41 studies were included, 35 with adult patients (N = 4 543 906) and 6 with caregivers of pediatric patients (N = 7921). Most studies (n = 38) were set in the US, and examined use of virtual care (n = 35). Adult patients facing language barriers had no significant difference in adjusted pooled odds of virtual vs in-person primary or specialist care use (adjusted OR [AOR], 0.91 [95% CI, 0.61-1.35]; n = 5; *I*^2^ = 95.0%) and significantly lower adjusted pooled odds of video vs telephone primary or specialist care use (AOR, 0.66 [95% CI, 0.52-0.85]; n = 5, *I*^2^ = 93.5%), with high heterogeneity. Restricting to specialist care, adult patients facing language barriers had significantly lower adjusted pooled odds of using virtual vs in-person (AOR, 0.78 [95% CI, 0.70-0.87]; n = 4; *I*^2^ = 0.0%) and video vs telephone (AOR, 0.62 [95% CI, 0.53-0.73]; n = 3; *I*^2^ = 0.0%) care, with low heterogeneity. Results for caregivers of pediatric patients were limited and showed no significant difference in odds of virtual vs in-person specialist care, with a wide confidence interval and high heterogeneity (OR, 0.62 [95% CI, 0.38-1.02]; n = 3; *I*^2^ = 91.2%). Results specific to primary care and results on other use of virtual care outcomes (eg, visit noncompletion) were inconclusive due to limited numbers of studies (eg, n = 2 studies reporting AOR of visit noncompletion for primary and specialist care, with *I*^2^ = 75.5% and 89.6%, respectively, for the corresponding meta-analyses). Results on satisfaction with virtual care, synthesized narratively, were limited and mixed. In studies of adult patients and caregivers of pediatric patients (n = 3 each), 2 of the 3 studies found no statistically significant difference in satisfaction, while 1 study found significantly lower satisfaction.

**Conclusions and Relevance:**

In this systematic review and meta-analysis, adult patients facing language barriers had no significant difference in pooled odds of using virtual compared with in-person care overall, and significantly lower pooled odds of using video compared to telephone care overall. Restricting to specialist care, pooled odds of using virtual care and video care were significantly lower among adult patients facing language barriers, with low heterogeneity. Further research on virtual care among individuals facing language barriers is needed, focusing on virtual primary care, patient satisfaction, and caregivers of pediatric patients.

## Key Points

**Question:**

Is there an association between language barriers and the use of or satisfaction with virtual care among adult patients and caregivers of pediatric patients in high-income countries?

**Findings:**

In this systematic review and meta-analysis of 41 studies and more than 4.5 million participants, adult patients facing language barriers had no statistically significant difference in adjusted pooled odds of virtual vs in-person care use overall (examining primary and specialist care), and significantly lower adjusted pooled odds of video vs telephone care use overall, with high heterogeneity. Restricting to specialist care, adjusted pooled odds of using virtual care and video care were significantly lower among adult patients facing language barriers, and heterogeneity was low.

**Meaning:**

Adults facing language barriers may experience inequities in use of virtual vs in-person specialist care and with video vs telephone specialist care.

## Teaser

This systematic review and meta-analysis examines the association between language barriers and use of and satisfaction with virtual care among adult patients and among caregivers of pediatric patients in high-income countries

## Introduction

Virtual care rapidly expanded following the onset of the COVID-19 pandemic, both in use and in available modalities, and is now an important and evolving mode of health care delivery. Its potential benefits include improved access to care for patients in rural areas, timeliness of care, and cost-effectiveness.^1^ Potential dangers include exacerbation of existing health inequities. Language barriers are a well-established determinant of health inequities, including longer hospital lengths of stay, poorer quality of care, and dissatisfaction with care,^2,3,4,5^ and there is rising evidence that these inequities may extend to virtual care. Qualitative studies from the US have indicated that virtual care models, especially those involving video platforms, have been challenging to navigate for patients facing language barriers due to absence of instructions for platform use in languages other than English and lack of access to or familiarity with required technology.^6,7^ Privacy concerns, inaccessibility of patient portals, and difficulty integrating interpreters into video visits could further compromise the accessibility and quality of virtual visits.^6,8,9^

Research on the association between language barriers and use of and satisfaction with virtual care in high-income countries grew considerably during the COVID-19 pandemic. There is now a substantial evidence base in this area examining different virtual care modalities, settings, and outcomes. To our knowledge, there has not yet been a systematic synthesis of this evidence. We conducted a systematic review and meta-analysis to synthesize the literature examining the association between language barriers and (1) virtual care use and (2) satisfaction with virtual care among adult patients and caregivers of pediatric patients in high-income countries.

## Methods

This review was reported following the Preferred Reporting Items for Systematic Reviews and Meta-analyses (PRISMA) reporting guideline 2020 guidance.^10^ A protocol was developed before initiating the review and is registered on PROSPERO (CRD42023426161). As this was a systematic review of previously published, publicly accessible data, this study was exempt from ethics review.

### Eligibility Criteria

We included quantitative, comparative studies of adults (aged 18 or older) living in high-income countries (as defined by the World Bank).^11^ Adult caregivers of pediatric patients were considered separately from adult patients given the different nature of health care visits as a caregiver of a child vs as the primary patient. Eligible studies compared groups with and without language barriers. Complete criteria for defining adult patients, caregivers of pediatric patients, and language barriers are presented in the eAppendix in Supplement 1.

There were 2 primary outcomes: (1) use of virtual care and (2) satisfaction with virtual care among individuals with and without language barriers. Virtual care consisted of outpatient telephone visits, video visits, or secure messaging between patients and a health care professional. Eligible measures of virtual care use included the proportion of patients who used virtual vs in-person outpatient care, ever vs never used virtual care, used one virtual care modality vs another, did not complete vs completed scheduled virtual care visits, and the number or rates of virtual care visits. Eligible measures of satisfaction included mean patient satisfaction scores or the proportion of individuals satisfied vs not satisfied with care, measured using patient satisfaction questionnaires. Eligible studies reported quantitative arm-level or contrast-level data specific to 1 or more eligible outcomes. Conference abstracts and letters to the editor were excluded.

### Data Sources and Search Strategy

We searched MEDLINE, Embase, and PsycINFO via Ovid and Web of Science via Clarivate from inception to March 10, 2023. We also conducted a gray literature search in April 2023 using Policy Commons, FedSys, Canadian Research Index, and OpenGrey. The database search strategies were designed by a health information specialist (E.U.) and included a combination of language and telehealth terms (eTables 1-4 in Supplement 1).

### Study Selection

References identified in the search were uploaded to and deduplicated in Covidence (Veritas Health Innovation) and then screened by 2 independent reviewers (E.W. and either Z.M. or S.M.G.) by title and abstract followed by full text. A sample of the eligibility criteria summary sheet used is presented in eTable 5 in Supplement 1. Full-text disagreements were resolved by consensus.

### Data Collection

Data were extracted by 2 independent reviewers (E.W. and either Z.M. or S.M.G.) using a piloted data extraction form in Covidence. Disagreements were resolved by consensus. Extracted variables included (1) study characteristics (title, first author, year of publication, country, study design, data source, outcome measures, virtual care types evaluated, virtual care setting, interpreter availability, and details on choice of care modality); (2) sample characteristics (overall sample size, language barrier definition, number and proportion facing or not facing language barriers, and languages spoken); and (3) aforementioned outcomes of interest, including all unadjusted and adjusted data along with corresponding measures of uncertainty.

### Risk of Bias Assessment

Risk of bias assessment was performed in duplicate by 2 independent reviewers (E.W. and Z.M. or D.D.) using the ROBINS-E tool.^12^ Age, sex, race and ethnicity, immigration recency, and health care need were considered important potential confounders. A piloted form in Covidence was used. Disagreements between reviewers were resolved by consensus. Eligibility for inclusion in the review was not reconsidered based on risk of bias assessments.

### Statistical Analysis

For both use and satisfaction with virtual care, results for adult patients and caregivers of pediatric patients were synthesized separately. For results on use of virtual care, log odds ratios were computed for meta-analyses. Separate meta-analyses were conducted for unadjusted and adjusted measures of association using random-effects models with the DerSimonian-Laird method; reporting was focused on the pooled adjusted results. For studies that reported adjusted results exclusively within language subgroups (eg, Spanish vs English, other vs English), the largest subgroup was included in the meta-analysis. Results were synthesized by outcome type, and subgroup analyses were conducted by type of care delivered (specialist vs primary). Results were summarized using odds ratios (ORs) and 95% CIs and were presented using forest plots. Heterogeneity was assessed using the *I*^2^ statistic. Reasons for heterogeneity were explored through prespecified subgroup analyses by type of care (specialist vs primary). Analyses were conducted using OpenMeta[analyst] for Windows 10 (64-bit).^13^ A 2-sided *P* < .05 was considered statistically significant. Results on satisfaction with virtual care were synthesized narratively due to limited studies and outcome heterogeneity.

## Results

### Search Results

**Figure 1.**
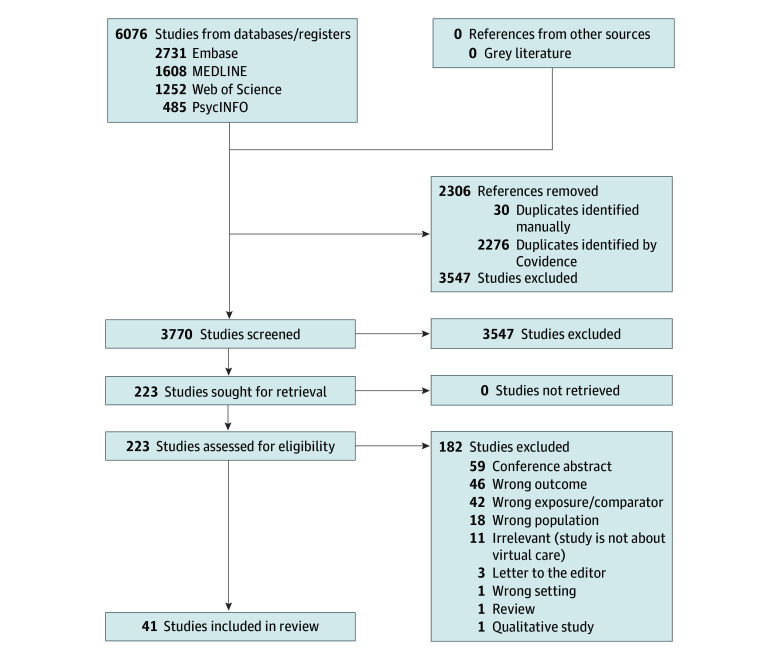
PRISMA Flow Diagram

Our database search produced 3770 unique records (Figure 1). After title and abstract screening, 3547 studies were excluded. In total, 223 studies were assessed for eligibility in full text, of which 41^14,15,16,17,18,19,20,21,22,23,24,25,26,27,28,29,30,31,32,33,34,35,36,37,38,39,40,41,42,43,44,45,46,47,48,49,50,51,52,53,54^ (35 with adult patients [N = 5 898 823 using study unit of analysis (visit or unique participant); N = 4 543 906 when limited to studies reporting N of unique participants]^14,15,16,17,18,19,20,21,22,23,24,25,26,27,28,29,30,31,32,33,34,35,36,37,38,39,40,41,42,43,44,45,46,47,48^ and 6 with caregivers of pediatric patients [N = 21 174 using study unit of analysis (visit or participant); N = 7921 when limited to studies reporting N of unique participants.]^49,50,51,52,53,54^) met eligibility criteria and were included in the review.

### Study Characteristics

#### Adult Patients

Most studies were from the US (n = 33),^14,15,16,17,18,19,20,21,22,23,24,25,26,27,28,29,30,31,34,35,36,37,38,39,40,41,42,43,44,45,46,47,48^ were set during the COVID-19 pandemic (n = 33),^14,15,16,17,18,19,20,21,22,23,24,25,26,27,28,29,30,31,32,33,34,35,36,37,38,39,40,43,44,45,46,47,48^ and examined use of virtual care (n = 32)^14,15,16,17,18,19,20,21,22,23,24,25,26,27,28,29,30,31,32,33,34,35,36,37,38,39,40,41,42,43,44,45^ (eTables 6-10 in Supplement 1). There were 3 studies of satisfaction with virtual care^46,47,48^ (eTable 11 in Supplement 1), all of which were cross-sectional surveys and examined a particular specialty. Most studies of virtual care use were cross-sectional or retrospective cohort studies using electronic medical records from a hospital or hospital network (n = 17)^17,18,19,22,23,24,25,26,29,32,33,34,35,37,38,43,44^ and reported on specialist care (n = 19).^18,19,20,21,22,23,24,25,26,27,28,32,33,36,37,38,43,44,45^ The remainder reported across both primary and specialty care (n = 11)^14,15,16,17,29,30,34,39,40,41,42^ or primary care exclusively (n = 2).^31,35^

Language barriers were identified in most studies using medical record or administrative database documentation of a preferred or primary language other than English (n = 17),^15,16,17,18,19,23,24,25,26,27,29,32,34,35,37,38,43^ and the remainder were identified using documented need for an interpreter in medical records or administrative databases (n = 6),^14,28,30,31,44,48^ self-report of a preferred or primary language or language spoken at home other than English (n = 3),^40,46,47^ self-reported low English ability (n = 3),^39,41,42^ a combination of documentation of primary or preferred language other than English and need for an interpreter in medical records (n = 2),^20,22^ use of a language other than English for surveys or assessments (n = 2),^21,33^ and primary language other than English with an unreported data source (n = 2).^36,45^ Among studies that reported the primary or preferred language of individuals facing language barriers, Spanish was the most represented.

#### Caregivers of Pediatric Patients

Most studies were from the US (n = 5)^49,50,51,53,54^ and were set during the COVID-19 pandemic (n = 5).^49,50,52,53,54^ There were 3 studies assessing the use of virtual care^49,50,51^ (eTable 12 in Supplement 1) and 3 studies assessing satisfaction^52,53,54^ (eTable 13 in Supplement 1). Studies on the use of virtual care were cross-sectional and reported on specialist care. Studies on patient satisfaction were cross-sectional surveys. Two surveys examined a particular specialty,^53,54^ and 1 examined across primary and specialist care.^52^ Language barriers were identified using documentation of a preferred or primary language other than English (n = 3),^50,53,54^ documented need for an interpreter (n = 1),^49^ self-report of a main language spoken at home other than English (n = 1),^52^ and a combination of documentation of primary language other than English and need for an interpreter (n = 1).^51^

#### Risk of Bias Assessment

In total, 38 included studies^14,15,16,17,19,20,21,22,23,24,25,26,27,28,29,30,31,32,33,34,35,36,37,38,39,40,41,42,43,44,45,47,48,49,50,51,53,54^ were rated at high overall risk of bias, and 3^18,46,52^ at very high overall risk of bias (eFigure 1 in Supplement 1). The high risk of bias ratings across included studies were predominantly due to confounding—all but 2 studies^15,33^ were rated at high risk of bias from confounding. Most did not adjust for confounders (n = 22),^17,18,20,21,22,24,25,26,30,32,36,38,39,43,45,47,48,50,51,52,53,54^ while others did not adjust for all predetermined important confounders (n = 17).^14,16,19,23,27,28,29,31,34,35,37,40,41,42,44,46,49^ Of studies that adjusted for confounders, most were missing adjustment for health care need (eg, comorbidity level) or immigration recency.

### Use of Virtual Care Among Adult Patients

#### Use of Virtual vs In-Person Care

**Figure 2.**
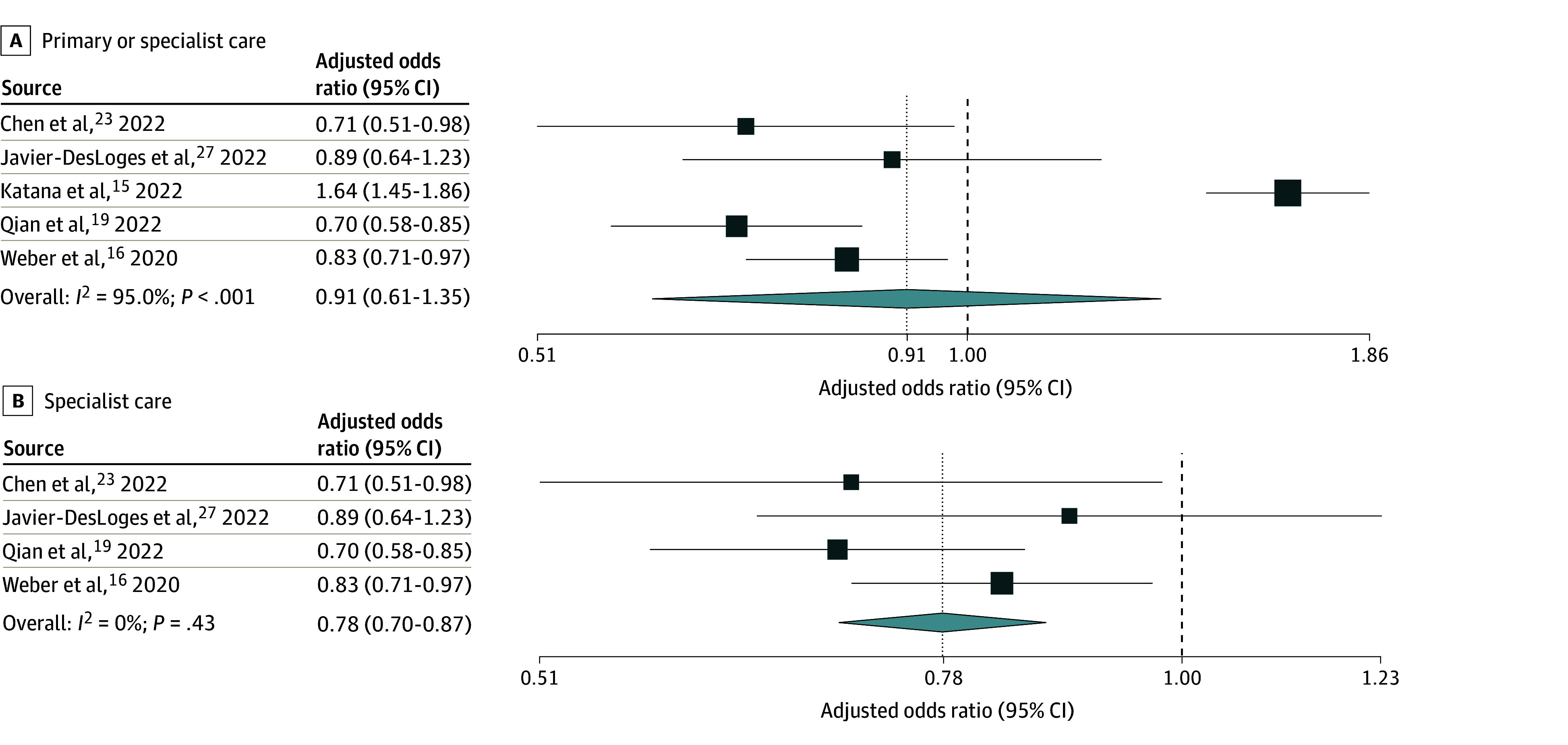
Random-Effects Meta-Analysis of Included Studies of Adult Patients Facing vs Not Facing Language Barriers That Reported Adjusted Odds Ratios of Use of Virtual vs In-Person Care Odds ratios are presented on a logarithmic scale. The vertical dotted line in each plot indicates the pooled odds ratio estimate. The vertical dashed line indicates an odds ratio of 1. Box sizes are proportional to the weight of each study. Horizontal lines indicate the 95% CI. *P* is the *P*-value for heterogeneity.

**Figure 3.**
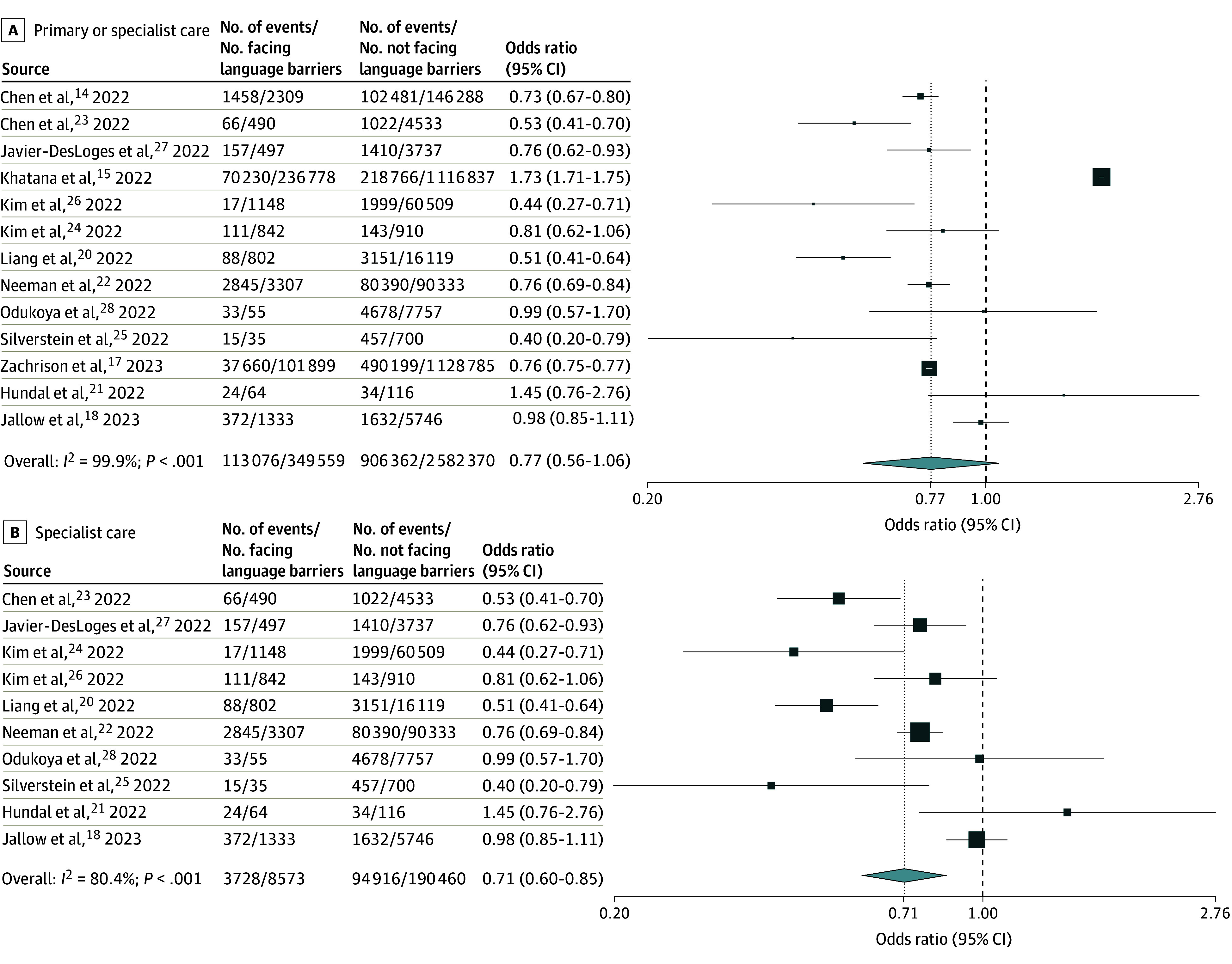
Random-Effects Meta-Analysis of Included Studies of Adult Patients Facing vs Not Facing Language Barriers That Reported Unadjusted Odds of Use of Virtual vs In-Person Care Odds ratios are presented on a logarithmic scale. The vertical dotted line in each plot indicates the pooled odds ratio estimate. The vertical dashed line indicates an odds ratio of 1. Box sizes are proportional to the weight of each study. Horizontal lines indicate the 95% CI. *P* is the *P*-value for heterogeneity.

A total of 15 studies^14,15,16,17,18,19,20,21,22,23,24,25,26,27,28^ (eTable 14 in Supplement 1) reported on the use of virtual vs in-person primary or specialist care in adult patients. Adults with vs without language barriers had no significant difference in adjusted pooled odds of using virtual primary or specialist care, with a wide confidence interval and high heterogeneity (adjusted OR [AOR], 0.91 [95% CI, 0.61-1.35]; n = 5; *I*^2^ = 95.0%) (Figure 2A). Results focused on specialist care showed significantly lower adjusted odds of virtual care use, with low heterogeneity (AOR, 0.78 [95% CI, 0.70-0.87]; n = 4; *I*^2^ = 0.0%) (Figure 2B). Pooled unadjusted odds for specialist care were similar, but with higher heterogeneity (OR, 0.71 [95% CI, 0.60-0.85]; n = 10; *I*^2^ = 80.4%) (Figure 3B). Meta-analysis of unadjusted results on primary or specialist care (n = 13 studies, including n = 10 of specialist care) again showed similar pooled odds, but with a wide, nonsignificant confidence interval and high heterogeneity (Figure 3A). No studies examined use of virtual vs in-person primary care exclusively.

#### Use of Video vs Telephone Visits

**Figure 4.**
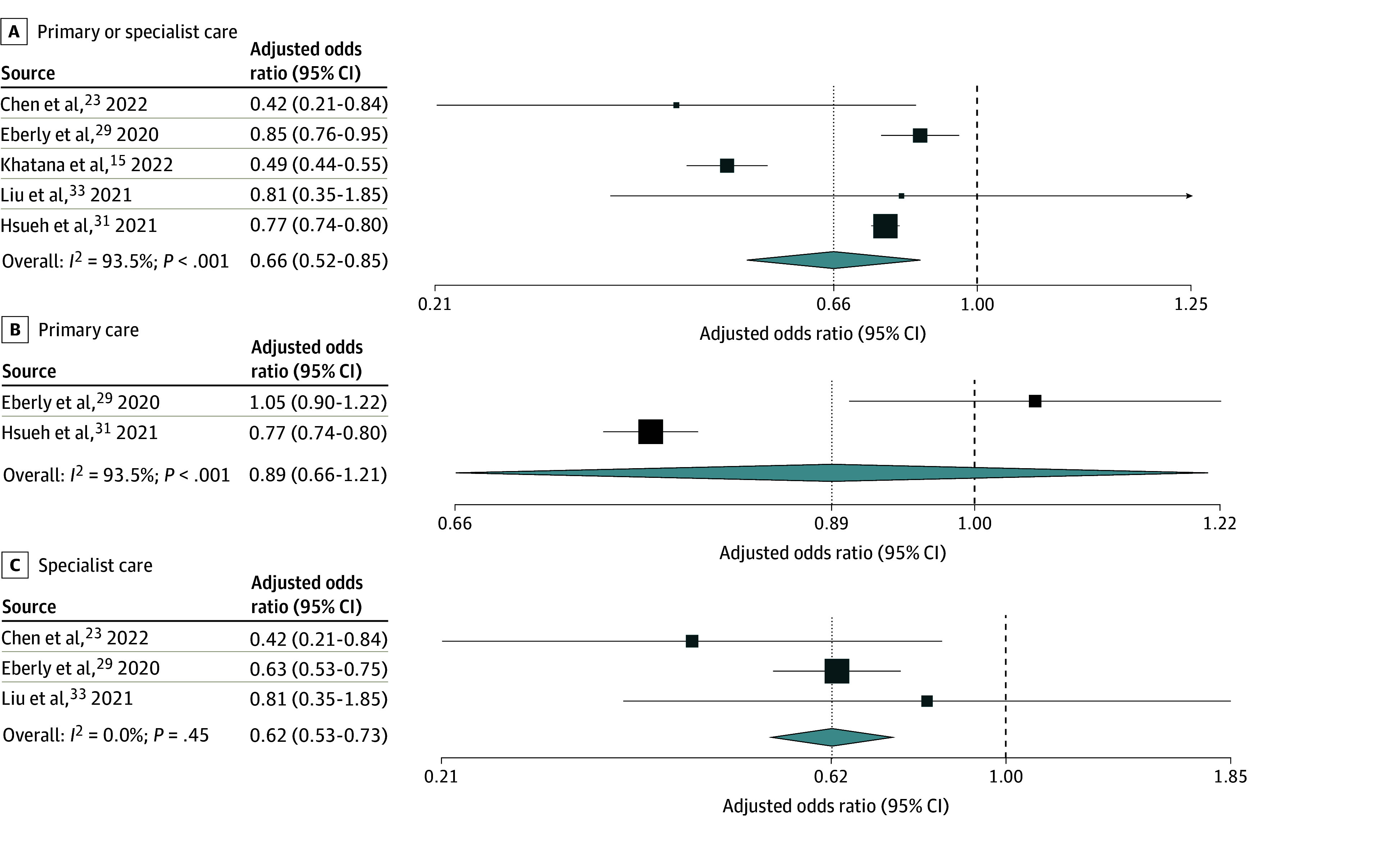
Random-Effects Meta-Analysis of Included Studies of Adult Patients Facing vs Not Facing Language Barriers That Reported Adjusted Odds Ratios of Video vs Telephone Visit Use Odds ratios are presented on a logarithmic scale. The vertical dotted line in each plot indicates the pooled odds ratio estimate. The vertical dashed line indicates an odds ratio of 1. Box sizes are proportional to the weight of each study. Horizontal lines indicate the 95% CI. *P* is the *P*-value for heterogeneity.

**Figure 5.**
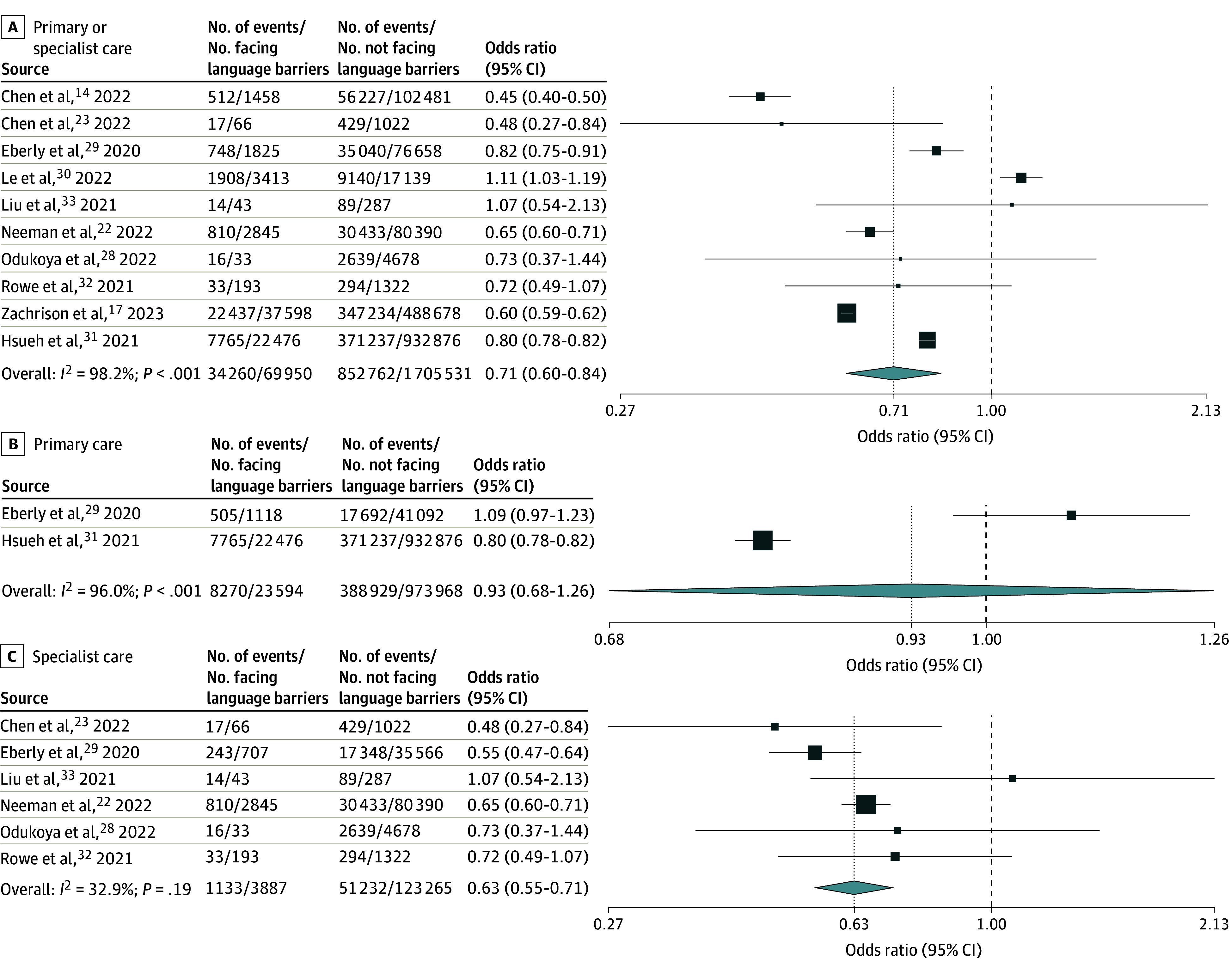
Random-Effects Meta-Analysis of Included Studies of Adult Patients Facing vs Not Facing Language Barriers That Reported Unadjusted Odds of Video vs Telephone Visit Use Odds ratios are presented on a logarithmic scale. The vertical dotted line in each plot indicates the pooled odds ratio estimate. The vertical dashed line indicates an odds ratio of 1. Box sizes are proportional to the weight of each study. Horizontal lines indicate the 95% CI. *P* is the *P*-value for heterogeneity.

Eleven studies^14,15,17,22,23,28,29,30,31,32,33^ (eTable 15 in Supplement 1) reported on video vs telephone use for primary or specialist care visits in adult patients. Adults with vs without language barriers had significantly lower adjusted pooled odds of video use, with high heterogeneity (AOR, 0.66 [95% CI, 0.52-0.85]; n = 5; *I*^2^ = 93.5%) (Figure 4A). Results focused on primary care showed no significant difference in adjusted odds of video use, with high heterogeneity (AOR, 0.89 [95% CI, 0.66-1.21]; n = 2; *I*^2^ = 93.5%) (Figure 4B). Results focused on specialist care showed significantly lower adjusted odds of video use, with low heterogeneity (AOR, 0.62 [95% CI, 0.53-0.73]; n = 3; *I*^2^ = 0.0%) (Figure 4C). Pooled unadjusted odds for specialist care were similar, but with higher heterogeneity (OR, 0.63 [95% CI, 0.55-0.71]; n = 6; *I*^2^ = 32.9%) (Figure 5C). Meta-analyses of unadjusted results on primary or specialist care and on primary care alone showed similar findings to meta-analyses of adjusted results (Figure 5A and Figure 5B).

#### Noncompletion of Scheduled Virtual Care Visits

Seven studies^26,29,34,35,36,37,38^ (eTable 16 in Supplement 1) reported on noncompletion vs completion of scheduled virtual visits among adult patients facing or not facing language barriers. Pooled adjusted odds of virtual primary or of specialist care visit noncompletion were similar between groups, but conclusions could not be drawn due to high heterogeneity (AOR, 1.02 [95% CI, 0.89-1.18]; n = 3; *I*^2^ = 89.0%) (eFigure 2A in Supplement 1). Meta-analyses of adjusted results focused on primary care (AOR, 0.99 [95% CI, 0.90-1.09]; n = 2; *I*^2^ = 75.5%) and specialist care (AOR, 1.11 [95% CI, 0.63-1.97; n = 2; *I*^2^ = 89.6%) were similarly limited (eFigure 2B and 2C in Supplement 1). Meta-analyses of unadjusted results were also inconclusive (eFigure 3 in Supplement 1).

#### Ever vs Never Use of Virtual Care

Six studies^30,39,40,41,42,43^ (eTable 17 in Supplement 1) reported on ever vs never use of virtual primary or specialist care. Adults facing language barriers had no significant difference in pooled adjusted odds of ever use of virtual primary and specialist care, with low heterogeneity (AOR, 0.84 [95% CI, 0.66-1.08]; n = 2; *I*^2^ = 0.0%) (eFigure 4 in Supplement 1). Meta-analysis of unadjusted results was inconclusive due to high heterogeneity and a wide confidence interval (eFigure 5 in Supplement 1).

#### Other Use of Virtual Care Outcomes

Four studies^22,23,44,45^ (eTable 18 in Supplement 1) examined additional outcomes on use of virtual care among adult patients. These showed lower odds of use of video visits compared with telephone or in-person visits alone and of only virtual compared with any in-person visits, as well as a lower median number of secure messages sent and received among adults facing language barriers.

#### Use of Virtual Care Among Caregivers of Pediatric Patients

Three studies^49,50,51^ (eTable 19 in Supplement 1) reported on use of virtual care in caregivers of pediatric patients, all examining use of virtual vs in-person specialist care. One study reported significantly lower adjusted odds of use of virtual care (AOR, 0.68 [95% CI, 0.59-0.78]).^49^ Meta-analysis of unadjusted results showed no statistically significant difference in odds of use of virtual care, with a wide confidence interval and high heterogeneity (OR, 0.62 [95% CI, 0.38-1.02]; n = 3; *I*^2^ = 91.2%) (eFigure 6 in Supplement 1).

### Satisfaction With Virtual Care Among Adult Patients

Three studies^46,47,48^ (eTable 20 in Supplement 1) reported on satisfaction with virtual care among adult patients. One study found significantly lower satisfaction with video and telephone rheumatology encounters among adults facing language barriers (top 2 box scores 58.3% vs 75.2%, *P* = .001), but no significant difference in preference for a virtual vs in-person visit (raw values not reported; *P* = .09); this study defined language barriers based on need for an interpreter.^48^ The remaining 2 studies used self-report of a preferred or primary language other than English and found nonsignificantly higher satisfaction, the first with video and telephone otolaryngology care on 2 satisfaction questionnaires (adjusted β coefficients 5.66 [95% CI, −6.03 to 17.36] and 0.27 [95% CI, −0.66 to 1.20)^46^ and the second with telehealth prenatal care (median [IQR] score, 23.0 [20.0-25.0] vs 22.0 [20.2-26.0]; *P* = .69).^47^

### Satisfaction With Virtual Care Among Caregivers of Pediatric Patients

Three studies^52,53,54^ reported on satisfaction with virtual care among caregivers of pediatric patients (eTable 21 in Supplement 1), all of which did not report adjusted results and defined language barriers based on documentation of a preferred or primary language or main language spoken at home other than English. One study found significantly lower satisfaction with video-based orthopedic surgery care (mean [SD] overall score, 3.40 [0.47] vs 3.72 [0.33]; *P* = .02).^54^ The second study found similar responses across 12 questions on video consultation satisfaction, save for a question on preference for in-person over telehealth care for their child (top 2 box responses 77.8% vs 61.4%).^52^ The third study found no significant differences in responses to 9 satisfaction questions on telephone and video surgery visits; incidence rate ratios for top box responses ranged from 0.84 (95% CI, 0.21-2.27) to 1.33 (95% CI, 0.40-3.25).^53^

## Discussion

Our systematic review and meta-analysis found that, among adult patients facing and not facing language barriers, use of virtual compared to in-person care was not significantly different overall (examining both primary and specialist care). However, restricting to specialist care, use of virtual care was significantly lower among adult patients facing language barriers, with low heterogeneity. We also found significantly lower use of video compared to telephone care among adult patients facing language barriers, both overall and within specialist care, with reduced heterogeneity when restricting to specialist care. Results on other use of virtual care outcomes, caregivers of pediatric patients, and satisfaction were inconclusive due to small numbers of studies, wide confidence intervals, or high heterogeneity.

Our results are consistent with literature describing barriers experienced by individuals facing language barriers in accessing virtual care. Following the onset of the COVID-19 pandemic, qualitative studies described difficulties experienced by patients facing language barriers with navigating platforms required for video-based virtual visits due to instructions being only in English.^6,7^ In a survey examining barriers to telehealth use among residents of New York public housing, lack of materials in patients’ preferred language was reported as a barrier by 22% of those surveyed and was associated with significantly reduced odds of telehealth use.^55^ Health care professionals have also described difficulty providing virtual care to patients with language barriers due to challenges associated with incorporating interpreters into video visits.^9,56,57^ Importantly, individuals facing language barriers may experience additional barriers to lack of language concordance in the video visit process; lack of access to required technology, limited digital literacy (more commonly among older adults), and prohibitive data or internet requirements have also been described as challenges in accessing video visits by individuals facing language barriers.^6,58^ Avenues for increasing the accessibility of virtual care for individuals facing language barriers should be explored, particularly for video visits (eg, options for instructions and patient portals in languages other than English, ensuring availability of interpreters).^8,58^ However, our findings also highlight the importance of maintaining access to telephone visits for those who cannot or do not wish to use video virtual visits.

Restricting the analysis to studies of specialist care led to substantial reductions in heterogeneity in our larger meta-analyses, indicating dissimilarity between results for primary care and specialist care. Studies focused on primary care were limited, and pooled results were inconclusive; some studies of virtual primary care use found that individuals facing vs not facing language barriers had higher use of virtual care, while other studies found the opposite. This finding suggests not only that use of virtual care among individuals facing language barriers may vary between specialist care and primary care, but also that use within primary care may contain additional differences. Future research is needed to confirm this potential variation.

As with any health system innovation, the expanded use of virtual care must be paired with understanding its uptake among marginalized groups to ensure that any inequities are understood and mitigated.^59^ In our review, studies on satisfaction with virtual care were limited and had mixed results; however, 2 studies showed evidence for lower satisfaction among individuals facing language barriers. Satisfaction with virtual care among individuals facing language barriers should be further explored, pairing quantitative and qualitative research to enable deeper exploration of determinants of satisfaction and areas for improvement. Improved knowledge in this regard will help ensure virtual care is equitably delivered to individuals facing language barriers.

### Limitations

Our review has limitations. First, risk of bias across included studies was high due to lack of adjustment for any or for all potential confounders. While we presented meta-analyses restricted to studies with confounder adjustment, most of these lacked adjustment for health care need or immigration recency. Thus, the results of our adjusted meta-analyses should be interpreted with potential residual confounding in mind. Second, some of our meta-analyses (eg, on primary care and caregivers, on virtual visit noncompletion) were limited by a small number of studies and high heterogeneity; more research is needed in these areas. Third, 38 of our included studies (93%) were set in the United States; therefore, our results are not likely generalizable to other countries. Fourth, most studies defined language barriers based on documentation of a preferred or primary language other than English (most commonly from electronic medical records), and the degree to which this represents a language barrier could vary on an individual basis. This may have led to underestimation of the magnitude of observed associations and contributed to heterogeneity in our meta-analyses. Researchers using electronic medical record data of preferred language should interpret results with this potential lack of consistency in mind. Fifth, the decision of whether to use virtual care can be guided by patient choice or clinician discretion, and the influence of language barriers on use of virtual care could differ accordingly. While we extracted available data on who or what guided the choice to use virtual care, not all studies reported this information, and we did not consider it in our analyses.

## Conclusions

In this systematic review and meta-analysis of the association between language barriers and the use of and satisfaction with virtual care among adults in high-income countries, adult patients facing language barriers had similar odds of receiving virtual care overall but lower pooled odds of using virtual compared with in-person specialist care, and of using video compared with telephone specialist care. Studies were primarily set in the US. Our findings highlight a gap in the delivery of virtual care to patients facing language barriers that may be present in other high-income countries. Future research should explore potential reasons for this gap and how it could be addressed. Further research is also needed on the use of virtual primary care, satisfaction with virtual care, and caregivers. In addition, future studies should control for all potential confounders; a more consistent definition of language barriers is also needed.

## Notes

Supplement 1. **eAppendix.** Additional eligibility criteria **eTable 1.** Search strategy for MEDLINE ALL (1946 to March 09, 2023) via Ovid **eTable 2.** Search strategy for Embase (1974 to 2023 Week 09) via Ovid **eTable 3.** Search strategy for APA PsycINFO (1806 to February Week 4, 2023) via Ovid **eTable 4.** Search strategy for Web of Science Core Collection via Clarivate **eTable 5.** Summary of study eligibility criteria used by reviewers **eTable 6.** Characteristics of included studies (n=15) of adult patients facing and not facing language barriers that compared use of virtual and in-person care, by specialty **eTable 7.** Characteristics of included studies (n=11) of adult patients facing and not facing language barriers that compared use of video and telephone visits, by specialty **eTable 8.** Characteristics of included studies (n=7) of adult patients facing and not facing language barriers that compared non-completion and completion of scheduled virtual care visits, by specialty **eTable 9.** Characteristics of included studies (n=6) of adult patients facing and not facing language barriers that compared ever vs never use of virtual care, by specialty **eTable 10.** Characteristics of included studies (n=4) of adult patients facing and not facing language barriers that examined other virtual care use outcomes **eTable 11.** Characteristics of included studies (n=3) of adult patients facing and not facing language barriers that examined satisfaction with virtual care, by specialty **eTable 12.** Characteristics of included studies (n=3) of caregivers of pediatric patients facing and not facing language barriers that examined use of virtual compared to in-person care, by specialty **eTable 13.** Characteristics of included studies (n=3) of caregivers of pediatric patients facing and not facing language barriers that examined satisfaction with virtual care, by specialty **eTable 14.** Summary of results across studies (n=15) of adult patients facing and not facing language barriers that examined use of virtual versus in-person care **eTable 15.** Summary of results across studies (n=11) of adult patients facing and not facing language barriers that examined use of video versus telephone visits **eTable 16.** Summary of results across studies (n=7) of adult patients facing and not facing language barriers that examined non-completion versus completion of scheduled virtual care visits **eTable 17.** Summary of results across studies (n=6) of adult patients facing and not facing language barriers that examined ever versus never use of virtual care **eTable 18.** Summary of results across studies (n=4) of adult patients facing and not facing language barriers that examined other use of virtual care outcomes **eTable 19.** Summary of results across studies (n=3) of caregivers of pediatric patients facing and not facing language barriers that examined use of virtual versus in-person care **eTable 20.** Summary of results across studies (n=3) of adult patients facing and not facing language barriers that examined satisfaction with virtual care **eTable 21.** Summary of results across studies (n=3) of caregivers of pediatric patients facing and not facing language barriers that examined satisfaction with virtual care **eFigure 1.** ROBINS-E risk of bias assessment of included studies (n=41) **eFigure 2.** Random effects meta-analysis of included studies of adult patients facing and not facing language barriers that reported adjusted odds ratios of non-completion vs completion of scheduled virtual care visits **eFigure 3.** Random effects meta-analysis of included studies of adult patients facing and not facing language barriers that reported unadjusted odds of non-completion vs completion of scheduled virtual care visits **eFigure 4.** Random effects meta-analysis of included studies (n=2) of adult patients facing and not facing language barriers that reported adjusted odds ratios of ever versus never use of virtual primary and specialist care **eFigure 5.** Random effects meta-analysis of included studies (n=4) of adult patients facing and not facing language barriers that reported unadjusted odds of ever versus never use of virtual primary and/or specialist care **eFigure 6.** Random effects meta-analysis of included studies (n=3) of caregivers of pediatric patients facing and not facing language barriers that reported unadjusted odds of use of virtual versus in-person specialist care

Supplement 2.
Data Sharing Statement
