# Supplementary material for: Virtual Care Among Adults Facing Language Barriers: A Systematic Review and Meta-Analysis
Source: JAMA Netw Open. 2025 Jun 5;8(6):e2513906. doi: 10.1001/jamanetworkopen.2025.13906 (PMC12142448; doi:10.1001/jamanetworkopen.2025.13906)
Supplement: Supplement 1. — eAppendix. Additional eligibility criteria eTable 1. Search strategy for MEDLINE ALL (1946 to March 09, 2023) via Ovid eTable 2. Search strategy for Embase (1974 to 2023 Week 09) via Ovid eTable 3. Search strategy for APA PsycINFO (1806 to February Week 4, 2023) via Ovid eTable 4. Search strategy for Web of Science Core Collection via Clarivate eTable 5. Summary of study eligibility criteria used by reviewers eTable 6. Characteristics of included studies (n=15) of adult patients facing and not facing language barriers that compared use of virtual and in-person care, by specialty eTable 7. Characteristics of included studies (n=11) of adult patients facing and not facing language barriers that compared use of video and telephone visits, by specialty eTable 8. Characteristics of included studies (n=7) of adult patients facing and not facing language barriers that compared non-completion and completion of scheduled virtual care visits, by specialty eTable 9. Characteristics of included studies (n=6) of adult patients facing and not facing language barriers that compared ever vs never use of virtual care, by specialty eTable 10. Characteristics of included studies (n=4) of adult patients facing and not facing language barriers that examined other virtual care use outcomes eTable 11. Characteristics of included studies (n=3) of adult patients facing and not facing language barriers that examined satisfaction with virtual care, by specialty eTable 12. Characteristics of included studies (n=3) of caregivers of pediatric patients facing and not facing language barriers that examined use of virtual compared to in-person care, by specialty eTable 13. Characteristics of included studies (n=3) of caregivers of pediatric patients facing and not facing language barriers that examined satisfaction with virtual care, by specialty eTable 14. Summary of results across studies (n=15) of adult patients facing and not facing language barriers that examined use of virtual versus in-perso [file jamanetwopen-e2513906-s001.pdf]

Supplementary Online Content

Wennberg E, Mohmand Z, D'Arienzo D, et al. Virtual care among adults facing language barriers: a systematic review and meta-analysis. *JAMA Netw Open*. 2025;8(6):e2513906. doi:10.1001/jamanetworkopen.2025.13906

eAppendix. Additional eligibility criteria

eTable 1. Search strategy for MEDLINE ALL (1946 to March 09, 2023) via Ovid

eTable 2. Search strategy for Embase (1974 to 2023 Week 09) via Ovid

eTable 3. Search strategy for APA PsycINFO (1806 to February Week 4, 2023) via Ovid

eTable 4. Search strategy for Web of Science Core Collection via Clarivate

eTable 5. Summary of study eligibility criteria used by reviewers

eTable 6. Characteristics of included studies (n=15) of adult patients facing and not facing language barriers that compared use of virtual and in-person care, by specialty

eTable 7. Characteristics of included studies (n=11) of adult patients facing and not facing language barriers that compared use of video and telephone visits, by specialty

eTable 8. Characteristics of included studies (n=7) of adult patients facing and not facing language barriers that compared non-completion and completion of scheduled virtual care visits, by specialty

eTable 9. Characteristics of included studies (n=6) of adult patients facing and not facing language barriers that compared ever vs never use of virtual care, by specialty

eTable 10. Characteristics of included studies (n=4) of adult patients facing and not facing language barriers that examined other virtual care use outcomes

eTable 11. Characteristics of included studies (n=3) of adult patients facing and not facing language barriers that examined satisfaction with virtual care, by specialty

eTable 12. Characteristics of included studies (n=3) of caregivers of pediatric patients facing and not facing language barriers that examined use of virtual compared to in-person care, by specialty

eTable 13. Characteristics of included studies (n=3) of caregivers of pediatric patients facing and not facing language barriers that examined satisfaction with virtual care, by specialty

eTable 14. Summary of results across studies (n=15) of adult patients facing and not facing language barriers that examined use of virtual versus in-person care

eTable 15. Summary of results across studies (n=11) of adult patients facing and not facing language barriers that examined use of video versus telephone visits

© 2025 Wennberg E et al. *JAMA Network Open*.

eTable 16. Summary of results across studies (n=7) of adult patients facing and not facing language barriers that examined non-completion versus completion of scheduled virtual care visits

eTable 17. Summary of results across studies (n=6) of adult patients facing and not facing language barriers that examined ever versus never use of virtual care

eTable 18. Summary of results across studies (n=4) of adult patients facing and not facing language barriers that examined other use of virtual care outcomes

eTable 19. Summary of results across studies (n=3) of caregivers of pediatric patients facing and not facing language barriers that examined use of virtual versus in-person care

eTable 20. Summary of results across studies (n=3) of adult patients facing and not facing language barriers that examined satisfaction with virtual care

eTable 21. Summary of results across studies (n=3) of caregivers of pediatric patients facing and not facing language barriers that examined satisfaction with virtual care

eFigure 1. ROBINS-E risk of bias assessment of included studies (n=41)

eFigure 2. Random effects meta-analysis of included studies of adult patients facing and not facing language barriers that reported adjusted odds ratios of non-completion vs completion of scheduled virtual care visits

eFigure 3. Random effects meta-analysis of included studies of adult patients facing and not facing language barriers that reported unadjusted odds of non-completion vs completion of scheduled virtual care visits

eFigure 4. Random effects meta-analysis of included studies (n=2) of adult patients facing and not facing language barriers that reported adjusted odds ratios of ever versus never use of virtual primary and specialist care

eFigure 5. Random effects meta-analysis of included studies (n=4) of adult patients facing and not facing language barriers that reported unadjusted odds of ever versus never use of virtual primary and/or specialist care

eFigure 6. Random effects meta-analysis of included studies (n=3) of caregivers of pediatric patients facing and not facing language barriers that reported unadjusted odds of use of virtual versus in-person specialist care

This supplementary material has been provided by the authors to give readers additional information about their work.

eAppendix. Additional eligibility criteria

Population definition: Studies with a mixture of adult and pediatric patients were eligible for inclusion as a study of adult patients provided that a minimum of 80% of the sample were adults. For studies reporting virtual care use, those exclusively including pediatric patients (<18 years) were considered studies of caregivers of pediatric patients, since caregivers (and their role in choosing visit modality) would likely be needed for visits with most children. For studies reporting satisfaction with virtual care, the patient population had to be specified as including only caregivers of pediatric patients. Mixed adult and pediatric samples were not permitted for studies of caregivers of pediatric patients, to prevent further bias from that already present due to older children (e.g., 16-17) possibly scheduling and attending visits alone. Exceptions were made for studies with mixed samples which reported results specific to an eligible population.

Exposure and comparator definitions: There is to our knowledge no recognized global gold standard for defining or identifying language barriers in healthcare, though a definition based on preference for a language other than English in healthcare settings has been recommended in the United States.[1] We included the following frequently used definitions: having a primary or preferred language that is not the (or an) official language of an individual's country of residence; having "low proficiency" in the official language(s) of the country of residence; or need for an interpreter. We did not include studies that examined whether an interpreter was used at the visit at which outcome measurement occurred, because of the potential for results on use of virtual care to be strongly influenced by differences in interpreter availability between modalities and because individuals who needed but did not receive an interpreter for their visit would be assigned to the no language barrier group. There was otherwise no restriction on the method of language barrier identification (e.g., self-report, language assessment, administrative records).

References

[1] Linggonegoro DW, Sanchez-Flores X, Huang JT. How telemedicine may exacerbate disparities in patients with limited English proficiency. J Am Acad Dermatol. Jun 2021;84(6):e289-e290. doi:10.1016/j.jaad.2021.02.032

© 2025 Wennberg E et al. *JAMA Network Open*.

eTable 1. Search strategy for MEDLINE ALL (1946 to March 09, 2023) via Ovid

Search Number	Description	Results
1	Language/	50,834
2	communication barriers/ or limited english proficiency/	7,319
3	((english or language) adj2 proficien*).ti,ab,kf.	3,684
4	"non English speaker*".ti,ab,kf.	234
5	(foreign adj2 language adj2 speaker*).ti,ab,kf.	27
6	(second adj2 language).ti,ab,kf.	3,293
7	("non-native" adj2 speaker*).ti,ab,kf.	353
8	language arts/ or multilingualism/ or translating/	11,555
9	(interpretor* or interpreter*).mp.	4,349
10	(linguistic* or multilingual or translat*).mp.	475,588
11	(language adj2 barrier*).mp.	3,016
12	(communication adj2 barrier*).mp.	8,713
13	or/1-12	529,387
14	telemedicine/ or remote consultation/ or telenursing/ or telepathology/ or teleradiology/ or telerehabilitation/	43,649
15	(teletherap* or telehealth or telemed* or e-health or ehealth or telerehab* or telehab* or telecar* or telepract* or telepsych* or (virtual adj2 care)).mp.	66,099
16	(remote adj2 consult*).mp.	6,574
17	(remote adj2 access).mp.	1,066
18	(video adj2 consult*).mp.	1,481
19	or/14-18	72,777
20	13 and 19	1,608

Search date: 2023-03-10

© 2025 Wennberg E et al. *JAMA Network Open*.

eTable 2. Search strategy for Embase (1974 to 2023 Week 09) via Ovid

Search Number	Description	Results
1	Language/	109,606
2	communication barriers/ or limited english proficiency/	3,693
3	((english or language) adj2 proficien*).ti,ab,kf.	4,562
4	"non English speaker*".ti,ab,kf.	511
5	(foreign adj2 language adj2 speaker*).ti,ab,kf.	28
6	(second adj2 language).ti,ab,kf.	3,189
7	("non-native" adj2 speaker*).ti,ab,kf.	408
8	language arts/ or multilingualism/ or translating/	112,911
9	(interpretor* or interpreter*).mp.	6,351
10	(linguistic* or multilingual or translat*).mp.	578,606
11	(language adj2 barrier*).mp.	4,666
12	(communication adj2 barrier*).mp.	5,208
13	or/1-12	676,305
14	telemedicine/ or remote consultation/ or telenursing/ or telepathology/ or teleradiology/ or telerehabilitation/	59,004
15	(teletherap* or telehealth or telemed* or e-health or ehealth or telerehab* or telehab* or telecar* or telepract* or telepsych* or (virtual adj2 care)).mp.	81,641
16	(remote adj2 consult*).mp.	1,848
17	(remote adj2 access).mp.	1,517
18	(video adj2 consult*).mp.	2,134
19	or/14-18	95,445
20	13 and 19	2,731

Search date: 2023-03-10

© 2025 Wennberg E et al. *JAMA Network Open*.

eTable 3. Search strategy for APA PsycINFO (1806 to February Week 4, 2023) via Ovid

Search Number	Description	Results
1	Language/	43,500
2	communication barriers/ or limited english proficiency/	6,733
3	((english or language) adj2 proficien*).ti,ab,id.	7,267
4	"non English speaker*".ti,ab,id.	127
5	(foreign adj2 language adj2 speaker*).ti,ab,id.	52
6	(second adj2 language).ti,ab,id.	13,828
7	("non-native" adj2 speaker*).ti,ab,id.	887
8	language arts/ or multilingualism/ or translating/	6,140
9	(interpretor* or interpreter*).mp.	3,837
10	(linguistic* or multilingual or translat*).mp.	145,790
11	(language adj2 barrier*).mp.	1,837
12	(communication adj2 barrier*).mp.	3,531
13	or/1-12	199,475
14	telemedicine/ or remote consultation/ or telenursing/ or telepathology/ or teleradiology/ or telerehabilitation/	7556
15	(teletherap* or telehealth or telemed* or e-health or ehealth or telerehab* or telehab* or telecar* or telepract* or telepsych* or (virtual adj2 care)).mp.	13,088
16	(remote adj2 consult*).mp.	831
17	(remote adj2 access).mp.	138
18	(video adj2 consult*).mp.	246
19	or/14-18	13,681
20	13 and 19	485

Search date: 2023-03-10

© 2025 Wennberg E et al. *JAMA Network Open*.

eTable 4. Search strategy for Web of Science Core Collection via Clarivate

Search Number	Description	Results
1	TI = (((english or language) NEAR/2 proficien*) OR "non English speaker" OR (foreign NEAR/2 language NEAR/2 speaker*) OR (second NEAR/2 language) OR ("non-native" NEAR/2 speaker*) OR interpretor* OR interpreter* OR linguistic* OR multilingual OR translat* OR (language NEAR/2 barrier*) OR (communication NEAR/2 barrier*)) OR AB = (((english or language) NEAR/2 proficien*) OR "non English speaker" OR (foreign NEAR/2 language NEAR/2 speaker*) OR (second NEAR/2 language) OR ("non-native" NEAR/2 speaker*) OR interpretor* OR interpreter* OR linguistic* OR multilingual OR translat* OR (language NEAR/2 barrier*) OR (communication NEAR/2 barrier*)) OR KP = (((english or language) NEAR/2 proficien*) OR "non English speaker" OR (foreign NEAR/2 language NEAR/2 speaker*) OR (second NEAR/2 language) OR ("non-native" NEAR/2 speaker*) OR interpretor* OR interpreter* OR linguistic* OR multilingual OR translat* OR (language NEAR/2 barrier*) OR (communication NEAR/2 barrier*))	949,139
2	TI = (teletherap* OR telehealth OR telemed* OR e-health OR ehealth OR telerehab* OR telehab* OR telecar* OR telepract* OR telepsych* OR (virtual NEAR/2 care) OR (remote NEAR/2 consult*) OR (remote NEAR/2 access) OR (video NEAR/2 consult*)) OR AB = (teletherap* OR telehealth OR telemed* OR e-health OR ehealth OR telerehab* OR telehab* OR telecar* OR telepract* OR telepsych* OR (virtual NEAR/2 care) OR (remote NEAR/2 consult*) OR (remote NEAR/2 access) OR (video NEAR/2 consult*)) OR KP = (teletherap* OR telehealth OR telemed* OR e-health OR ehealth OR telerehab* OR telehab* OR telecar* OR telepract* OR telepsych* OR (virtual NEAR/2 care) OR (remote NEAR/2 consult*) OR (remote NEAR/2 access) OR (video NEAR/2 consult*))	63,714
3	1 and 2	1,252

Search date: 2023-03-10

© 2025 Wennberg E et al. *JAMA Network Open*.

eTable 5. Summary of study eligibility criteria used by reviewers

PICO term	Inclusion	Exclusion
Population	Sample is composed entirely of adult (18+) patients or caregivers of pediatric patients who are residents of high-income countries<br><br>OR<br><br>sample is mixed (e.g., both adult and pediatric patients) but study reports eligible outcome data specific to the population of interest.	Sample is not composed of patients or caregivers (e.g., a study of healthcare providers)<br><br>OR<br><br>sample is composed exclusively of child (<18) patients or of adult patients/caregivers who are residents of low-income countries<br><br>OR<br><br>sample is mixed but no data specific to the population of interest is reported.
Exposure	Study includes a language barrier* group.<br><br>* Language barrier defined as primary/preferred language other than the official language(s) of the country of residence (self-reported or collected through administrative records), low proficiency in official language(s) of the country of residence (self-reported, collected through administrative records, or measured through language test), or need for an interpreter	Does not include a language barrier group.
Comparator	Study includes a no language barrier group.	Does not include a no language barrier group.
Outcomes	Reports quantitative data on:<br><br>1) use of virtual care in individuals with compared to without language barriers, measured as:<br>a. Number or rates of virtual care visits in each group<br>b. Proportion in each group who used vs did not use virtual care (eligible comparators for virtual care use include: no virtual care visits, in person visits, ER visits)<br>c. Proportion in each group who used one virtual care modality vs another virtual care modality (e.g., telephone visits vs video visits)	Study examines an eligible virtual care intervention, but no data is reported on either primary outcome<br><br>OR<br><br>outcome measure is not eligible:<br><br>• Includes studies which report the proportion of individuals with vs without language barriers who used virtual care, but do not report the proportion in each group who did not use VC (e.g.,

© 2025 Wennberg E et al. *JAMA Network Open*.

	d. Proportion of non-attended virtual care appointments in each group<br>and/or<br>2) Patient satisfaction with virtual care in individuals with language barriers compared to individuals without language barriers, measured as:<br>a. Mean or median scores in each group on a patient satisfaction scale<br>b. Proportion satisfied vs not satisfied (over/under a certain threshold on a patient satisfaction scale) in each group<br><br>Eligible virtual care interventions include <u>telephone visits, video visits, and secure messaging through patient portals</u> between patients and healthcare providers.	studies describing demographic changes in the patient population using VC throughout the pandemic)<br>OR<br>Study describes use or satisfaction with an ineligible intervention, including:<br>• Mobile-based patient education interventions (e.g., educational apps)<br>• Remote patient monitoring interventions, unless data is reported specific to an eligible intervention component (e.g., telephone visits that are part of the larger remote monitoring intervention)
Study design	Quantitative, comparative study. No restriction by retrospective vs prospective.	Non-comparative study (e.g., case report), qualitative study, review, letter to the editor.
Publication type	No restriction by publication date or language.	Conference abstract.
Setting	Set in a high-income country (World Bank definition – see list at bottom of page: https://data.worldbank.org/country/XD)	Not set in a high-income country.

© 2025 Wennberg E et al. *JAMA Network Open*.

eTable 6. Characteristics of included studies (n=15) of adult patients facing and not facing language barriers that compared use of virtual and in-person care, by specialty

Study (population; specialty), Setting	Population (n)	Study period	Study design; data source	Recruitment	Virtual care modality; type of care examined	Selection of visit modality (patient and/or provider)	Interpreter available for visits	Language barrier definition	Language barrier group (n, %); language(s) spoken (n, %)	No language barrier group (n, %); language (s) spoken (n, %)	Outcome
Chen 2022a (primary/specialist), United States	Patients with outpatient encounters eligible for telehealth across all specialties at Michigan Medicine (148,997)	Apr 1 to Jun 30 2020	Cross-sectional; health administrative database (Epic Clarity)	Extracted all eligible patient encounters using scheduling and billing databases (Epic Clarity)	Telephone and video; outpatient care across all specialties	NR, telehealth provided by providers whenever possible	NR	Need for vs no need for interpreter documented in electronic medical record (missing n=400, 0.3%)	2309 (1.5); NR	146,288 (98.2); NR	Proportion of participants who received any video or telephone visits vs only in-person visits
Khatana 2022 (primary/specialist), United States	Adults (18 years+) with outpatient encounters during the COVID-19 pandemic across the continental US (1,999,534)	Mar 1 to Dec 31 2020	Cross-sectional; health administrative database (Healthjump)	Obtained medical encounter data from Healthjump electronic medical record data management platform	Telephone and video; all outpatient ambulatory care	NR	NR	Language other than English vs English as primary language in electronic medical records (missing n=645,919, 32.3%)	236,778 (11.8); Spanish (21.2), other: (78.8)	1,116,837 (55.9); English (100)	Proportion of participants with any video or telephone encounter vs in-person only encounters

© 2025 Wennberg E et al. *JAMA Network Open*.

Weber 2020 (primary/specialist – COVID-related care), United States	First encounters for voluntary COVID-19 testing or care via telehealth, ER, or office visit within the Mount Sinai system (39,299)	Mar 20 to May 18 2020	Cross-sectional; health administrative database (Mount Sinai deidentified COVID-19 database)	Identified using Mount Sinai deidentified COVID-19 database	Video, telephone, and secure messaging; voluntary care or testing for COVID-19	NR	NR	Spanish vs English as preferred language in database; Language other than Spanish or English/unspecified language vs English as preferred language in database	3994 (10.2); Spanish: 2617 (6.7), Other/ unspecified: 1377 (3.5)	35,235 (89.8); English (100)	Proportion of outpatient office vs video/telephone/secure messaging first encounters; Proportion of ER vs video/telephone/secure messaging first encounters
Zachrison 2023 (primary/specialist), United States	Patients with at least one ambulatory care visit within the study period at a large New England healthcare system (1,241,313; pre-COVID: 935,938, COVID: 860,101)	Oct 1 2019 to Sep 30 2020; pre-COVID: Oct 1 2019 to Mar 14 2020, COVID: Mar 15 to Sep 30 2020	Cross-sectional; electronic medical records	Identified using encounter data from electronic health record system	Telephone and video; ambulatory care from any health care provider care (primary, specialty, and behavioral health)	Patient; based on patient preference or capability	NR	Language other than English vs English as preferred language in electronic health record (missing n=10,629, 0.9%; pre-COVID: 6876, 0.7%, COVID: 4483, 0.5%)	101,899 (8.2) (pre-COVID: 76,951 (8.2)<br>COVID: 65,710 (7.6)); NR	1,128,785 (90.9) (pre-COVID: 852,111 (91.0)<br>COVID: 789,908 (91.8)); English (100)	Proportion of participants with any telephone or video appointment vs only in-person

© 2025 Wennberg E et al. *JAMA Network Open*.

Jallow 2023 (specialist – dermatology), United States	Outpatient dermatology clinic visits at Georgetown University Hospital, Washington Hospital Center, and Chevy Chase prior to and during the COVID-19 pandemic (7,079; n unique participants NR)	Apr to Jun 2020 (quarantine period); Jan to Mar 2021 (post-quarantine period)	Cross-sectional; electronic medical records	Identified eligible visits using electronic medical records	Video; outpatient dermatology care	NR	NR	Non-English speaking or unknown vs English speaking, using electronic medical record	April to June 2020: 392<br>January to March 2021: 941	April to June 2020: 2090<br>January to March 2021: 3656	Proportion of visits that were video vs in-person
Qian 2022 (specialist – oncology), United States	Encounters of cancer patients over 18 with medical oncology physicians and involving survivorship/palliative care within the University of	Jan 1 to Sep 30 2020	Cross-sectional; electronic medical records	Identified all eligible encounters in the study period, presumably using electronic medical records but not explicitly stated	Telephone and video; medical oncology care (initial consultations with medical oncologists, on-treatment assessment, follow-up post-treatment encounters, visits involving	Provider; patient disease teams identified patients eligible for telemedicine	Yes	Language other than English vs English as preferred language in electronic medical record	3860 (13.1); Spanish: 2489 (64.5), other: 1371 (35.5)	25,561 (86.9); English: 25,561 (100)	Proportion of visits that were telephone or video vs in-person

© 2025 Wennberg E et al. *JAMA Network Open*.

	California San Diego Health System (29,421; n unique participants =8997)				survivorship/ palliative care)						
Liang 2022 (specialist – gynecologic oncology), United States	Gynecologic oncology visits Sutter Health in northern California (17,599; n unique participants NR)	Mar 2020 to Aug 2021	Cross-sectional; NR	NR	Telehealth; gynecologic oncology care	NR	NR	First language other than English and required interpreter services vs English as first language (missing n=678; 3.8%)	802 (4.7); NR	16,119 (95.3); NR	Proportion of visits that were telehealth vs in-person
Hundal 2022 (specialist – hematology-oncology), United States	Patients ≥18 with confirmed or suspected cancer or hematological disease served by cancer centres within an	May to Nov 2020	Cross-sectional; survey	Participants invited to complete survey in waiting or examination rooms before or after provider visit	Telephone and video; care for oncological or hematological disease during the COVID-19 pandemic	NR	NR	Use of Spanish vs English language survey	55; Spanish (100)	125; English 116 (92.8), English and Spanish: 6 (4.8), English and Other: 3 (2.4)	Proportion of participants who self-reported any video or telephone use vs in-person care alone for oncological

© 2025 Wennberg E et al. *JAMA Network Open*.

	integrated healthcare system in Connecticut who responded to questions regarding accessing care for oncological or hematological disease during the COVID-19 pandemic (180)							Language other than English vs English as self-reported primary language spoken at home	64; Spanish: 61 (95.3), other: 3 (4.7)	116; English (100)	or hematological disease during the COVID-19 pandemic
Neeman 2022 (specialist – hematology-oncology), United States	Outpatient encounters between a patient and a hematology and/or medical oncology physician or advance practice provider within the Kaiser Permanente Northern	Mar 19 to Sep 30 2020	Retrospective cohort; electronic medical records and US 2010 census data	Identified eligible encounters within study period using electronic health record data	Video and telephone; outpatient hematology or medical oncology consultations and return visits	Provider	Yes	Primary language other than English and need for interpreter vs no need for interpreter or unmarked field in electronic health record (missing	3307 (3.4); NR	90,333 (92.3); NR	Proportion of video/telephone vs in-person encounters

© 2025 Wennberg E et al. *JAMA Network Open*.

	California region (97,887; n unique participants = 46,052)							n=4247, 4.3%)			
Chen 2022b (specialist – ophthalmology), United States	Patients ≥18 who received outpatient ophthalmic care at the Yale New Haven Hospital health system during the first COVID-19 surge in the United States (5023)	Mar 1 to Aug 31 2020	Cross-sectional; electronic medical records	Identified all eligible encounters using electronic health records	Telephone and video; any appointment with an ophthalmology provider	Provider; based on clinical need for in-person encounter and patient ability to use telehealth	NR	Language other than English vs English as primary language in electronic health record	490 (9.8); NR	4533 (90.2); English (100)	Proportion of participants with at least 1 video or telephone visit vs in-person visits alone
Kim 2022a (specialist – physical therapy), United States	In-person and telehealth physical therapy visits at Duke Sports Sciences Institute, Durham, North	Mar 1 2020 to Dec 31 2021	Cross-sectional; electronic medical records	Identified visits from electronic health record database	Telephone and video; physical therapy for nonoperative (acute and chronic conditions) and postoperative rehabilitation	Pre-September 2020: Provider; virtual favoured, post-op patients had priority for in-person	NR	Primary language other than English vs English primary language in electronic medical record	1148 (1.9); NR	60,509 (98.1); English (100)	Proportion of visits that were telephone or video vs in-person

© 2025 Wennberg E et al. *JAMA Network Open*.

	Carolina (61,657; n unique participants =7892)					Post-September 2020: Patient					
Silverstein 2022 (specialist – minimally invasive gynecologic surgery), United States	Patients referred for any indication to the Minimally Invasive Gynecologic Surgery Division of the University of North Carolina Department of Obstetrics and Gynecology and seen by an MIGS provider in 2020 (753)	2020	Retrospective cohort; electronic medical records and internal referral database	Identified all eligible referred patients using internal referral database	Video; new Minimally Invasive Gynecologic Surgery consults referred for any indication	Patient; video appointments offered to all patients, in-person if there was need for physical exam, inability to use video appointments, or preference to be seen in person	Yes	Language other than English vs English as primary language in electronic medical record (missing n=18, 2.4%)	35 (4.6); Spanish: 33 (94.3), other: 2 (5.7)	700 (93.0); English: 700 (100)	Proportion of participants who received a video vs an in-person appointment following referral
Kim 2022b (specialist – otolaryngology head and neck surgery), United States	Patients >18 with completed otolaryngology head and neck surgery encounters	2020	Cross-sectional; medical records	Retrospective chart review	Telephone; otolaryngology head and neck surgery encounters	Provider; based on clinical need	NR	Language other than English vs English as primary language in	842 (48.1); Spanish: 442 (52.5), Cantonese: 209 (24.8), Other: 191 (22.7)	910 (51.9); English (100)	Proportion of participants who completed a telephone

© 2025 Wennberg E et al. *JAMA Network Open*.

	at an urban safety-net hospital in San Francisco, California (1752)							medical records			vs in-person visit
Javier-DesLoges 2022 (specialist – urology), United States	Visits among patients older than 18 years old seen for a urologic condition at UC San Diego Health (4234; n unique participants = 2516)	Mar 15 to Sep 30 2020	Cross-sectional; NR	Retrospectively identified (method NR)	Telephone and video; non-procedural urologic care visits	Provider; virtual offered to all patients, changed based on provider opinion of appropriateness	NR	Language other than English vs English as preferred language in electronic medical record	497 (11.7); Spanish: 349 (70.2), Other: 148 (29.8)	3737 (88.3); English (100)	Proportion of visits that were telephone or video vs in-person
Odukoya 2022 (specialist – urology), United States	Outpatients with urological encounters at Michigan Medicine (7851)	Apr 1 to Jun 30 2020	Cross-sectional; health administrative database (Epic Clarity)	Identified all eligible encounters using scheduling and billing database	Video and telephone; urological patient encounters (evaluation and management visits, including postoperative visits)	Provider	Yes	Need for interpreter vs no need for interpreter documented in database (missing n=39 0.5%)	55 (0.7); NR	7757 (98.8); NR	Proportion of participants with any video or telephone vs in-person only encounters

ER = emergency room; NR = not reported

eTable 7. Characteristics of included studies (n=11) of adult patients facing and not facing language barriers that compared use of video and telephone visits, by specialty

Study (population; specialty), Setting	Population (n)	Study period	Study design; data source	Recruitment	Virtual care modality; type of care examined	Selection of visit modality (patient and/or provider)	Interpreter available for visits	Language barrier definition	Language barrier group (n, %); language(s) spoken (n, %)	No language barrier group (n, %); language(s) spoken (n, %)	Outcome
Chen 2022a (primary/specialist), United States	Patients with telehealth outpatient encounters across all specialties at Michigan Medicine (104,204)	Apr 1 to Jun 30 2020	Cross-sectional; health administrative database (Epic Clarity)	Extracted all eligible patient encounters using Michigan Medicine scheduling and billing databases, Epic Clarity	Telephone and video; outpatient care across all specialties (evaluation and management visits, annual wellness visits, post-operative visits, and other visit types)	NR	NR	Need for vs no need for interpreter documented in electronic medical record (missing 265, 0.2%)	1458 (1.4); NR	102,481 (98.4); NR	Proportion of participants who received any video vs only telephone visits
Eberly 2020 (primary/specialist), United States	First telephone or video encounters within the study period for adult (18+) patients	Mar 16 to May 11 2020	Retrospective cohort; electronic medical records	Identified eligible patients using electronic medical records	Telephone and video; primary (general and family medicine) and specialty care	NR; seems telemedicine mandated and video potentially preferred	NR	Language other than English vs English as preferred language in electronic medical record	1825 (2.3); NR<br><br>primary care clinics subgroup: 1118 (2.6),	76,658 (97.6); English (100)<br><br>primary care clinics subgroup: 41,092	Proportion of participants seen via video vs telephone for their first encounter

© 2025 Wennberg E et al. *JAMA Network Open*.

	seen at one primary care or specialty ambulatory clinic within a large academic health system (78,539; primary care clinics subgroup: 42,242, specialty care clinics subgroup: 36,297)				(cardiology, pulmonology, rheumatology, gastroenterology, infectious diseases, rheumatology, nephrology, hematology-oncology)			(missing 56, 0.1%; primary care clinics subgroup: 32, 0.1%; specialty care clinics subgroup: 24, 0.1%)	specialty care clinics subgroup: 707 (1.9)	(97.3), specialty care clinics subgroup: 35,566 (98.0)	in the study period
Khatana 2022 (primary/specialist), United States	Adults (18 years+) with virtual outpatient encounters during the COVID-19 pandemic across the continental US (432,634)	Mar 1 to Dec 31 2020	Cross-sectional; health administrative database (Healthjump)	Obtained medical encounter data from Healthjump electronic medical record data management platform	Telephone and video; all outpatient ambulatory care	NR	NR	Language other than English vs English as primary language in electronic medical record	NR	NR	Proportion of participants with any video vs telephone-only encounters
Le 2022 (primary/specialist), United States	Adult (over 18) workers in Washington State with	Mar to Oct 2020	Cross-sectional; health administr	Administrative workers' compensation claims data	Telephone, video, and secure messaging; care related	For individuals who required interpreter	Yes	Ever vs never use of interpreter services in	3481 (16.8); NR	17,235 (83.2); NR	Proportion of participants with any video vs

© 2025 Wennberg E et al. *JAMA Network Open*.

	an accepted State Fund claim for a work-related injury/illness and at least one related, paid bill for virtual services in study period (20,716)		ative database		to a work injury/illness covered by worker's compensation agencies	services, seems provider: all appointments were arranged by health care and vocational providers		administrative billing data			only telephone or secure messaging vs secure messaging alone virtual encounters
Zachrison 2023 (primary/specialist), United States	Patients with at least one virtual ambulatory care visit within the study period at a large New England healthcare system (526,959)	Mar 15 to Sep 30 2020	Cross-sectional; electronic medical records	Identified using encounter data from<br>electronic health record system	Telephone and video; ambulatory care from any health care provider care (primary, specialty, and behavioral health)	Patient; based on patient preference or capability	NR	Language other than English vs English as preferred language in electronic health record (missing n=683, 0.1%)	37,598 (7.1); NR	488,678 (92.8); English (100)	Proportion of participants with any video vs telephone-only virtual visits
Hsueh 2021 (primary), United States	Primary care telemedicine visits self-scheduled via the patient	Mar 16 to Oct 31 2020	Cross-sectional; "automated data sources"	Identified all eligible visits within Kaiser Permanente Northern California	Telephone and video; primary care	Patient; based on patient preference or capability	NR	Need for interpreter vs no need for interpreter documented	22,476 (2.4); NR	932,876 (97.6); NR	Proportion of telemedicine visits scheduled

© 2025 Wennberg E et al. *JAMA Network Open*.

	portal at Kaiser Permanente Northern California (955,352; n unique participants =642,370)							in electronic health record			as video vs telephone
Rowe 2021 (specialist – cardiology), Australia	Outpatients of all cardiology clinics conducted at St Vincent's Hospital Melbourne who completed a telemedicine appointment (1515)	Mar 17 to Aug 12 2020	Cross-sectional; electronic medical records	Electronic medical records and Medicare billing codes from St Vincent's Hospital Melbourne	Telephone and video; outpatient cardiology care (initial consultations, review consultations, follow-up appointments)	Patient and provider; video as default with change to telephone based on patient or provider preference	Yes	Language other than English vs English as preferred language in electronic medical record	193 (12.7); NR	1322 (87.3); English (100)	Proportion of participants who used video vs telephone for their initial appointment in the study period
Liu 2021 (specialist – geriatric medicine), Canada	Patients who received at least one virtual assessment in the geriatric medicine clinic at St. Michael's	Mar 17 to Jul 13 2020	Cross-sectional; electronic medical records	Retrospective review of medical records	Telephone and video; geriatric medicine virtual assessment	Patient or caregiver	Yes	English vs language other than English as language of assessment	43 (13.0); NR	287 (87.0); English (100)	Proportion of participants who received a video vs telephone virtual assessment

© 2025 Wennberg E et al. *JAMA Network Open*.

	Hospital, Toronto (330)										
Neeman 2022 (specialist – hematology-oncology), United States	Virtual outpatient encounters between a patient and a hematology and/or medical oncology physician or advance practice provider within the Kaiser Permanente Northern California region (86,960; n unique participants NR)	Mar 19 to Sep 30 2020	Retrospective cohort; electronic medical records and US 2010 census data	Identified eligible encounters within study period using electronic health record data	Telephone and video; outpatient hematology or medical oncology consultations and return visits	Provider	Yes	Primary language other than English and need for interpreter vs no need for interpreter or unmarked field in electronic health record (missing n=3725, 4.3%)	2845 (3.3); NR	80390 (92.4); NR	Proportion of virtual visits that were video vs telephone
Chen 2022b (specialist – ophthalmology), United States	Patients ≥18 who received video or telephone outpatient ophthalmic care at the Yale New	Mar 1 to Aug 31 2020	Cross-sectional; electronic medical records	Identified all eligible encounters using electronic health records	Telephone and video; any appointment with an ophthalmology provider	Patient; video encouraged, phone used if patients unable to access or did not	NR	Language other than English vs English as primary language in electronic health record	66 (6.1); NR	1022 (93.9); English (100)	Proportion of participants with telephone visits alone vs any video visit

© 2025 Wennberg E et al. *JAMA Network Open*.

	Haven Hospital health system during the first COVID-19 surge in the United States (1088)					want to use video					
Odukoya 2022 (specialist – urology), United States	Outpatients with telehealth urological encounters at Michigan Medicine (4744)	Apr 1 to Jun 30 2020	Cross-sectional; health administrative database (Epic Clarity)	Identified all eligible encounters using scheduling and billing database	Video and telephone; urological patient encounters (evaluation and management visits, including postoperative visits)	Provider and patient	Yes	Need for interpreter vs no need for interpreter documented in database (missing n=33, 0.7%)	33 (0.7); NR	4678 (98.6); NR	Proportion of participants with any video vs only telephone visits

NR = not reported

© 2025 Wennberg E et al. *JAMA Network Open*.

eTable 8. Characteristics of included studies (n=7) of adult patients facing and not facing language barriers that compared non-completion and completion of scheduled virtual care visits, by specialty

Study (population; specialty), Setting	Population (n)	Study period	Study design; data source	Recruitment	Virtual care modality; type of care examined	Selection of visit modality (patient and/or provider)	Interpreter available for visits	Language barrier definition	Language barrier group (n, %); language(s) spoken (n, %)	No language barrier group (n, %); language (s) spoken (n, %)	Outcome
Eberly 2020 (primary/specialist), United States	First scheduled telephone or video encounters within the study period for adult (18+) patients at a primary care or specialty ambulatory clinic within a large academic health system (148,402; primary care clinics subgroup: 76,062, specialty	Mar 16 to May 11, 2020	Retrospective cohort; electronic medical records	Electronic medical record	Telephone and video visits; primary (general and family medicine) and medical specialty care (cardiology, pulmonology, rheumatology, gastroenterology, infectious diseases, rheumatology, nephrology, hematology-oncology)	NR; seems telemedicine mandated and video potentially preferred	NR	Language other than English vs English as preferred language in electronic medical record (missing n=408, 0.3%; primary care clinics subgroup 221, 0.3%; specialty care clinics subgroup 187, 0.3%)	3,895 (2.6)<br>primary care clinics subgroup: 2076 (2.7)<br>specialty care clinics subgroup: 1819 (2.5)	144,099 (97.1)<br>primary care clinics subgroup: 73,765 (97.0)<br>specialty care clinics subgroup: 70,334 (97.2)	Proportion of participants who completed vs who cancelled without rescheduling or no-showed a scheduled video or telephone encounter

© 2025 Wennberg E et al. *JAMA Network Open*.

	care clinics subgroup: 72,340)										
Gmunder 2021 (primary/specialist), United States	Scheduled telemedicine visits within University of Miami Health System (362,764; n unique participants NR)	Mar 1 to Oct 31 2020	Cross-sectional; electronic medical records, Internal Revenue Service 2018 income tax data	Identified all eligible patient visits from University of Miami Health System records	Video; primary/specialist care	NR	NR	Language other than English vs English as preferred language in electronic health record (missing n=1100, 0.3%)	101,950 (28.1); Spanish: 98,194 (96.3), Other: 3756 (3.7)	259,714 (71.6); English (100)	Proportion of completed vs non-completed scheduled video visits
Chen 2022c (primary), United States	Adult primary care telephone visits scheduled with physicians, nurse practitioners, and physician assistants at 23 adult primary care clinics at New York City Health + Hospitals (132,713; n	Mar 2020 to Jun 2020 ("telehealth transition period")	Cross-sectional; electronic health records	Identified all eligible visits using electronic health record data	Telephone; adult primary care visits with physicians, nurse practitioners, and physician assistants	Clinic staff; telephone mandated, exceptions granted on case-to-case basis	NR	Language other than vs English vs English as primary language in electronic health record	NR; Spanish or other	NR; English (100)	Proportion of non-attended vs attended scheduled telephone visits

© 2025 Wennberg E et al. *JAMA Network Open*.

	unique participants NR)										
	Adult primary care telephone and video visits scheduled with physicians, nurse practitioners, and physician assistants at 23 adult primary care clinics at New York City Health + Hospitals (telephone: 305,197; video: 26,232; n unique participants NR)	Jul 2020 to Aug 2021 (“elective telehealth period”)			Telephone and video; adult primary care visits with physicians, nurse practitioners, and physician assistants	Patient			NR; Spanish or other	NR; English (100)	Proportion of non-attended vs attended scheduled telephone visits; proportion of non-attended vs attended scheduled video visits
Pitaro 2022 (adult; specialist –	Patients scheduled for virtual education classes	Aug 2020 to Apr 2021	Retrospective cohort study, NR	NR (“prospectively collected data”)	Video; total joint arthroplasty	N/A – all participants scheduled for virtual	NR	Language other than English vs English as primary	153 (12.0); NR	1122 (88.0); English (100)	Proportion of participants who did not attend vs who attended

© 2025 Wennberg E et al. *JAMA Network Open*.

orthopedics), United States	after primary total hip or total knee arthroplasty at a large metropolitan health system (1275)				education classes	during study period		language spoken, NR			scheduled virtual total joint arthroplasty education classes
Kim 2022b (adult; specialist otolaryngology head and neck surgery), United States	Patients >18 with completed and missed otolaryngology head and neck surgery telephone encounters at an urban safety-net hospital in San Francisco, California (297)	2020	Cross-sectional; medical records	Retrospective chart review of all completed and missed telehealth encounters	Telephone; otolaryngology head and neck surgery encounters	Provider; based on “anticipated clinical need”	NR	Language other than English vs English as primary language in medical records	130 (43.8); Spanish: 75 (57.7), Cantonese: 28 (21.5), Other: 27 (20.8)	167 (56.2); English: 167 (100)	Proportion of participants who completed vs missed a telephone visit
Abou Ali 2023 (adult; specialist – vascular surgery), United States	Scheduled outpatient vascular surgery evaluations for patients over 18 across 12 outpatient	Feb 24 to Dec 31 2020	Cross-sectional; electronic medical records	Identified consecutive patients from institution's electronic medical records	Telephone and video; outpatient vascular surgery evaluation (follow-up	Patient and provider; provider assessed appropriateness of virtual and	Yes	Language other than English vs English spoken language in electronic	NR	NR; English (100)	Proportion of completed vs non-completed scheduled telephone and video visits

© 2025 Wennberg E et al. *JAMA Network Open*.

	vascular surgery sites in the Pittsburgh area (1559; n unique participants NR)				or new patient)	patient had to agree to virtual		medical record			
Bell 2022 (adult; specialist – urology), United States	Patients scheduled for telephone-based telehealth encounters in the Department of Urology at Zuckerberg San Francisco General Hospital (322; n unique participants NR)	Mar 17 2020 to subsequent 8 weeks	Cross-sectional; electronic medical records	Retrospectively identified all eligible encounters using electronic medical records	Telephone; new patient, follow-up, and post-operative urology visits with advanced practice providers, attendings, and residents	No choice; exclusive use of telephone visits during study period	Yes	Language other than English vs English as primary language in electronic medical record	130 (40.4); Arabic: 2 (1.5), Cantonese: 29 (22.3), Mandarin: 5 (3.9), Other: 13 (10.0), Russian: 7 (5.4), Spanish: 70 (53.8), Tagalog: 4 (3.1)	192 (59.6); English: 192 (100)	Proportion of non-attended vs attended scheduled telephone visits

NR = not reported

© 2025 Wennberg E et al. *JAMA Network Open*.

eTable 9. Characteristics of included studies (n=6) of adult patients facing and not facing language barriers that compared ever vs never use of virtual care, by specialty

Study (population; specialty), Setting	Population (n)	Study period	Study design; data source	Recruitment	Virtual care modality; type of care examined	Selection of visit modality (patient and/or provider)	Interpreter available for visits	Language barrier definition	Language barrier group (n, %); language(s) spoken (n, %)	No language barrier group (n, %); language(s) spoken (n, %)	Outcome
Andersen 2021 (primary/specialist), United States	Marshallese adults living in the continental United States and Hawaii (109)	Jul 27 to Nov 20 2020	Cross-sectional; survey	Marshallese community health workers (Arkansas); Facebook pages (nationwide)	Video; primary or specialist medical care	NR	NR	Self-reported English speaking ability of not very well vs very well or well	13 (11.9); NR	96 (88.1); English: 96 (100) (very well: 45; well: 51)	Proportion of participants who self-reported use vs no use of video visits
Chang 2022 (primary/specialist), United States	Medicare beneficiaries who accessed medical care and whose primary care provider offered telehealth appointments during the COVID-19 pandemic (5,644,	Oct to Nov 2020	Cross-sectional; survey (Medicare Current Beneficiary Survey COVID-19 Fall Supplement Public Use File)	Sampled from existing sample members from the 2016, 2017, 2018, and 2019 Medicare Current Beneficiary Survey panels	Telephone and video visits; any medical care	NR	NR	Self-reported language other than English spoken at home vs English spoken at home (missing n=5, 0.09%)	744 (13.2); NR	4,895 (86.7); English (100)	Proportion of participants who self-reported use of one or more vs no telephone or video visits since July 1, 2020

© 2025 Wennberg E et al. *JAMA Network Open*.

	analyzed 5,589)										
El-Toukhy 2020 (primary/specialist), United States	Adults (over 18 years of age) who had accessed a patient portal at least once in the last year (2151)	Jan 25 to May 5 2017; Jan 26 to May 2 2018	Cross-sectional; survey (H5C1 and H5C2)	Random selection of nonvacant residential addresses stratified by minority concentration (H5C1); Next Birthday Method to select adult within next household (H5C2)	Secure messaging; communication with health care provider or staff	NR	NR	Self-reported English ability of well, not well, or not at all vs very well	NR	NR	Proportion of participants who self-reported use vs non-use of secure messaging with health care provider and staff via patient portal
Le 2022 (primary/specialist), United States	Adult (over 18) workers in Washington State with an accepted State Fund claim for a work-related injury/illness and at least one related, paid medical bill in study	Mar to Oct 2020	Cross-sectional; health administrative database	Administrative workers' compensation claims data	Telephone, video, and secure messaging; care related to a work injury/illness covered by worker's compensation agencies	For individuals who required interpreter services, likely provider choice: all appointments were arranged by health care and vocational providers	Yes	Ever vs never use of interpreter services in administrative billing data	11,451 (13.0); NR	76,746 (87.0); NR	Proportion of participants with any vs no telephone, video, or secure messaging encounter

© 2025 Wennberg E et al. *JAMA Network Open*.

	period (88,197)										
Rodriguez 2021 (primary/specialist), United States	Respondents of the 2015-2018 adult California Health Interview Surveys (non-institutionalized California residents 18 and older) (84,419)	2015-2018	Cross-sectional; survey (2015 adult California Health Interview Surveys)	List-assisted random digit dial sampling of landlines and cellphones from 44 geographic strata + oversampling of underrepresented populations	Telephone and video; care from a doctor or health professional	NR	NR	"Limited English proficiency" (English speaking ability of not well or not at all, survey response) vs "English proficiency" (definition NR)	8063 (15); NR	76,356 (85); English (100)	Proportion who self-reported receipt vs no receipt of care from a doctor or health professional through a video or telephone conversation rather than an office visit
Ramsey 2022 (specialist – ophthalmology), United States	Patients with diabetes mellitus previously seen in an ophthalmology clinic in 2019 and not yet returned for eye examination by the start of the COVID-19	Mar 15 to Dec 31 2020	Cross-sectional; electronic medical record	Identified through electronic medical record	Telephone and video; ophthalmology eye care	Provider	NR	Language other than English vs English as primary language spoken recorded in electronic medical record	440 (5.4)	7707 (94.6); English (100)	Proportion of participants who used vs did not use a telephone or video in study period, limited to first visit in study period

© 2025 Wennberg E et al. *JAMA Network Open*.

	pandemic (8147)										

NR = not reported

© 2025 Wennberg E et al. *JAMA Network Open*.

eTable 10. Characteristics of included studies (n=4) of adult patients facing and not facing language barriers that examined other virtual care use outcomes

Study (population; specialty), Setting	Population (n)	Study period	Study design; data source	Recruitment	Virtual care modality; type of care examined	Selection of visit modality (patient and/or provider)	Interpreter available for visits	Language barrier definition	Language barrier group (n, %); language(s) spoken (n, %)	No language barrier group (n, %); language(s) spoken (n, %)	Outcome
Chen 2022b (specialist – ophthalmology), United States	Patients ≥18 who received outpatient ophthalmic care at the Yale New Haven Hospital health system during the first COVID-19 surge in the United States (5023)	Mar 1 to Aug 31 2020	Cross-sectional; electronic medical records	Identified all eligible encounters using electronic health records	Telephone and video; any appointment with an ophthalmology provider	Provider choice; based on need for in-person encounter and patient ability to use telehealth	NR	Language other than English vs English as primary language in electronic health record	490 (9.8); NR	4533 (90.2); English (100)	Proportion of participants with any video visit vs telephone alone or in-person alone
Neeman 2022 (specialist – hematology-oncology), United States	Patients with encounters with a hematology and/or medical oncology physician	Mar 19 to Sep 30 2020	Retrospective cohort; electronic medical records and US 2010	Identified eligible encounters within study period using electronic health record data	Secure messaging; outpatient hematology or medical oncology consultatio	NR	Yes	Primary language other than English and need for interpreter vs no need for interpreter	NR	NR	Median number (n, IQR) of secure messages sent and received per patient

© 2025 Wennberg E et al. *JAMA Network Open*.

	or advance practice provider within the Kaiser Permanente Northern California region (46,052)		census data		ns and return visits			or unmarked field in electronic health record			
Tong 2022 (specialist – oncology), United States	Oncology patients visiting the Froedtert Health Centre in Wisconsin (46,943)	Mar 2020 to Mar 2022	Retrospective cohort; electronic medical records	Used Clinical Research Data Warehouse database to collect Froedtert Health Centre patients' electronic health records Health Records	Telephone, video, and secure messaging; oncologic care	NR	Yes	Need for vs no need for interpreter documented in electronic medical record (missing n=478, 1.0%)	660 (1.4); NR	45,805 (97.6); NR	Proportion of participants who used any video vs in-person alone or in-person and telephone/text message; Proportion of participants who used any video vs only telephone/text message and/or in-person; Proportion of participants

© 2025 Wennberg E et al. *JAMA Network Open*.

											who used any telephone/text message vs only video and/or in-person; Proportion of participants who used any in-person vs only video and/or telephone/text message
Wood 2021 (specialist – HIV care), United States	Persons 18 and over with HIV who were engaged in care prior to and who completed at least one visit during the COVID-19 pandemic at an academic HIV clinic in	Mar 16 to Dec 31 2020	Cross-sectional; NR	NR	Telephone and video; outpatient HIV care (clinic visit or HIV RNA lab check)	Patient and provider; video encouraged, phone offered as back-up option	NR	Language other than English vs English as primary language	302 (13.5); Spanish: 120 (39.7), other: 182 (60.3)	1938 (86.5); English: 1938 (100)	Proportion of participants who completed any video visit vs only use of phone and/or in-person visits

© 2025 Wennberg E et al. *JAMA Network Open*.

	Seattle, Washington (2240)										

NR = not reported

© 2025 Wennberg E et al. *JAMA Network Open*.

eTable 11. Characteristics of included studies (n=3) of adult patients facing and not facing language barriers that examined satisfaction with virtual care, by specialty

Study (population; specialty), Setting	Population (n)	Study period	Study design; data source	Recruitment	Virtual care modality; type of care examined	Selection of visit modality (patient and/or provider)	Interpreter available for visits	Language barrier definition	Language barrier group (n, %); language(s) spoken (n, %)	No language barrier group (n, %); language(s) spoken (n, %)	Outcome; tool
Choi 2022 (adult; specialist – otolaryngology), United States	Patients over 18 years with telemedicine encounters at the Keck Hospital otolaryngology clinic during COVID-19 (212)	May 11 to Jul 9 2020	Cross-sectional; survey	Identified eligible patients via chart review; eligible patients were provided a survey link via email within 1 week following their visit	Telephone and video; otolaryngology clinic new patient visits, follow-up visits, and post-operative visits	Patient (for new patients) and provider (for existing patients); telemedicine recommended	NR	Language other than English vs English as self-reported preferred language (missing n=6, 2.8%)	3 (1.4); NR	203 (95.8); NR	Mean overall scores; Press Ganey survey and Telemedicine Satisfaction Questionnaire
Futterman 2020 (adult; specialist – prenatal care), United States	Patients who received at least one virtual and one in-person prenatal care visit at low-risk and high-risk prenatal	Mar 1 to May 1 2020	Cross-sectional; survey	Phone call to every eligible patient; one phone call maximum, in which survey questions were posed, maximum of 5 days post-appointment	Telehealth; prenatal care	NR	NR	Language other than English vs English as self-reported primary language	56 (54); Spanish (100)	48 (46); English (100)	Median overall scores; Short Assessment of Patient Satisfaction (SAPS)

© 2025 Wennberg E et al. *JAMA Network Open*.

	clinics (inner city safety hospital net setting) and were monitored throughout the pregnancy by a physician or a midwife (104)										
Mortezavi 2021 (adult; specialist – rheumatology), United States	Rheumatology patients at the Rochester Regional Health Rheumatology office with virtual encounters from May-June 2020 (359; 186 phone, 173 video)	May-June 2020	Cross-sectional; survey	Identified eligible patients using encounter data and contacted them via phone 2-6 weeks after the encounter. Used interpreters to survey participants with language barriers.	Telephone and video; rheumatology follow-up and new patient visits	NR	Yes	Need for interpreter vs no need for interpreter	24 (6.7); Spanish, Arabic, Nepali, Moldovan (NR)	335 (93.3); English (100)	Proportion with top 2 box vs neutral vs bottom 2 box scores on "I was satisfied with my telemedicine encounter"; Proportion who responded yes vs no to "I would have preferred an in-

© 2025 Wennberg E et al. *JAMA Network Open*.

											person visit instead of a telemedicine visit”

NR=not reported

© 2025 Wennberg E et al. *JAMA Network Open*.

eTable 12. Characteristics of included studies (n=3) of caregivers of pediatric patients facing and not facing language barriers that examined use of virtual compared to in-person care, by specialty

Study (population; specialty), Setting	Population (n)	Study period	Study design; data source	Recruitment	Virtual care modality; type of care examined	Selection of visit modality (patient and/or provider)	Interpreter available for visits	Language barrier definition	Language barrier group (n, %); language(s) spoken (n, %)	No language barrier group (n, %); language (s) spoken (n, %)	Outcome
Cahan 2022 (specialist – 6 pediatric subspecialties), United States	Pediatric outpatient visits in 6 high-volume pediatric subspecialties at Stanford Children's Health (13,582, analyzed 13,232; n unique participants NR)	Mar to Jun 2020	Cross-sectional; health administrative database (Stanford Research Repository Tool)	Identified eligible visits from database (Stanford Research Repository Tool)	Telephone and video; pediatric cardiology, nephrology, oncology, neurology, pulmonary and endocrinology care (new and established patients)	NR	Yes	Family request for interpreter support vs no family request for interpreter support in clinical encounter data	NR	NR	Proportion of completed visits that were telephone or video vs in-person
Vaughan 2022 (specialist – cardiology), United States	Established pediatric (1-17) cardiology outpatients at Texas Children's	Mar 6 to Dec 31 2020	Cross-sectional; NR	NR	Telemedicine; outpatient cardiology care	NR	Yes	Language other than English vs English as primary language	982 (18.2); Spanish: 914 (93.1), Other/unable to obtain: 68 (6.9)	4428 (81.8); English (100)	Proportion of participants with any telemedicine visit vs

© 2025 Wennberg E et al. *JAMA Network Open*.

	Hospital (5410)							(measure NR)			in-person only
Dai 2021 (specialist - otolaryngology), United States	Follow-up encounters for patients <18 years of age who underwent tonsillectomy or adenotonsillectomy between July 1, 2016 to June 1, 2018 at a single tertiary care pediatric hospital serving Maryland and nearby states (50; n unique participants = 29)	Jul 1 2016 to Sep 1 2018	Retrospective cohort; electronic medical records	Identified eligible patients using medical records. Sampled all patients with non-English speaking and every 10th patient with English-speaking caregivers.	Telephone; Otolaryngology healthcare encounters regarding complications and post-operative care following tonsillectomy or adenotonsillectomy	Caregiver; caregivers told in discharge instructions to call otolaryngology service for follow-up, but ultimately caregiver choice	NR	Non-English speaking (primary caregiver had primary language other than English and required or requested an interpreter in electronic medical record) vs English speaking	21 (42.0); NR	29 (58.0); English (100)	Proportion of visits to primary care provider vs telephone calls to otolaryngology service for follow-up; Proportion of ED visits vs telephone calls to otolaryngology service for follow-up

NR = not reported

© 2025 Wennberg E et al. *JAMA Network Open*.

eTable 13. Characteristics of included studies (n=3) of caregivers of pediatric patients facing and not facing language barriers that examined satisfaction with virtual care, by specialty

Study (population; specialty), Setting	Population (n)	Study period	Study design; data source	Recruitment	Virtual care modality; type of care examined	Selection of visit modality (patient and/or provider)	Interpreter available for visits	Language barrier definition	Language barrier group (n, %); language(s) spoken (n, %)	No language barrier group (n, %); language (s) spoken (n, %)	Outcome; tool
Hiscock 2022 (primary/specialist), Australia	Caregivers of pediatric patients who completed a video telehealth consultation at a tertiary pediatric hospital in Melbourne, Victoria (2436)	Jun 3 to Aug 25 2020	Cross-sectional; survey	Consecutively recruited via link to survey sent following the end of video consultation	Video; any video consultation at a tertiary pediatric hospital in Melbourne, Australia	NR	NR	Language other than English vs English as self-reported main language spoken at home (missing n=102, 4.2%)	238 (9.8); NR	2096 (86.0); English (100)	Proportion of participants with top 2 vs bottom 2 box responses on nine telehealth evaluation questions and five preferences for care questions
Cockrell 2022 (specialist - surgery), United States	Parents and caregivers of children who were seen by a surgical subspecialty physician	Apr 1 to Jun 30 2020	Cross-sectional; survey	All patients and caregivers were invited to complete the Family Experience Survey after their encounter via emailed	Telephone and video; outpatient pediatric surgical subspecialty encounters (cardiac,	Patient and provider; option of telephone, video, or in-person given when	Yes	Spanish vs English preferred language of care	NR, Spanish (100)	NR, English (100)	Proportion of top box responses (9 or 10 on 0-10 numeric scale; 4 on 4-point Likert

© 2025 Wennberg E et al. *JAMA Network Open*.

	or advanced practice provider via telephone or video (NR)			link or telephone call (English speakers) or telephone call (Spanish speakers)	general and thoracic, neurosurgery, ophthalmology, orthopedics, otolaryngology, plastics, transplant, urology);	considered appropriate by provider					scale); 9 questions from Family Experience Survey
Hanna 2022 (specialist – orthopedic surgery), United States	Caregivers of pediatric orthopedic surgery patients seen by a fellowship-trained pediatric orthopedic surgeon via telemedicine at an orthopedic clinic in New Jersey (46)	Mar 23 to Jun 3 2020	Cross-sectional; survey and patient charts	Caregivers invited to participate by the orthopedic surgeon after the video visit and subsequently contacted by phone to complete survey	Video; pediatric orthopedic surgery visits	N/A - all patients seen via telemedicine during study period	Yes	Primary language other than English vs primary language of English or bilingual including English in patient chart	11 (24); Spanish and Haitian Creole (100)	35 (76); English: 33 (94.3), bilingual English and Spanish: 2 (5.7)	Mean participant scores; 10 questions from Patient Satisfaction Questionnaire, rated on 4-point Likert scale (1=poor, 2=fair, 3=good, 4=excellent)

NR = not reported

© 2025 Wennberg E et al. *JAMA Network Open*.

eTable 14. Summary of results across studies (n=15) of adult patients facing and not facing language barriers that examined use of virtual versus in-person care

Study	Exposure, comparator	Outcome	Raw proportions (n, %)	Model, measure (covariates)	Inferential value
Chen 2022a (primary/ specialist)	Need for vs no need for interpreter documented in electronic health record	Proportion of participants who received any video or telephone visits vs only in-person visits	Telephone/video: 1458 (63.1) vs 102,481 (70.0)<br><br>In-person: 851 (36.9) vs 43,807 (30.0)[1]	N/A	N/A
Khatana 2022 (primary/ specialist)	**English vs language other than English** as primary language in electronic medical records	Proportion of participants with any telephone or video encounter vs in-person only encounters, complete case analysis (excluded n=645,919 participants with missing language and race/ethnicity)	Telephone/video: 218,766 (19.6) vs 70,230 (29.7)<br><br>In-person: 898,071 (80.4) vs 166,548 (70.3), p<0.001	Multivariable mixed effects logistic regression, OR [95% CI] (age; sex; race/ethnicity; comorbidities; proportion of rural residents living in the 3-digit ZIP code of residence; socioeconomic, household composition and disability, minority status and language index, and housing and transportation components of the social vulnerability index for the 3-digit ZIP code of residence; random intercepts for medical provider)	**0.61 (0.54-0.69), p<0.001**
		Proportion of participants with any telephone or video encounter vs in-person only encounters, multiple imputation	Telephone/video (proportion, SE): English: 77.9 (0.07) vs non-English: 22.1 (0.07)<br><br>In-person (proportion, SE):	Multivariable mixed effects logistic regression, OR [95% CI] (age; sex; race/ethnicity; comorbidities; proportion of rural residents living in the 3-digit ZIP code of residence; socioeconomic, household composition and disability, minority status and language index, and housing and transportation components of the social vulnerability index for the 3-digit	**0.78 (0.74-0.83), p<0.001**

© 2025 Wennberg E et al. *JAMA Network Open*.

			English: 84.3 (0.04) vs non-English: 15.7 (0.04)	ZIP code of residence; random intercepts for medical provider)	
Weber 2020 (primary/specialist – COVID-related care)	Spanish vs English as preferred language in database	Proportion of **outpatient office vs video/telephone/secure messaging** first encounters	NR	Multinomial logistic regression, OR [95% CI] (race/ethnicity, age, comorbidity sum)	**1.2 (1.03-1.4)**
		Proportion of **ER vs video/telephone/secure messaging** first encounters	NR	Multinomial logistic regression, OR [95% CI] (race/ethnicity, age, comorbidity sum)	**2.4 (2.1-2.6)**
	Language other than Spanish or English/unspecified language vs English as preferred language in database[2]	Proportion of **outpatient office vs video/telephone/secure messaging** first encounters	NR	Multinomial logistic regression, OR [95% CI] (race/ethnicity, age, comorbidity sum)	**1.1 (0.9-1.3)**
		Proportion of **ER vs video/telephone/secure messaging** first encounters	NR	Multinomial logistic regression, OR [95% CI] (race/ethnicity, age, comorbidity sum)	**2.5 (2.2-2.9)**
Zachrison 2023 (primary/ specialist)[2]	Language other than English vs English as preferred language in electronic health record	Proportion of participants who had at least one telephone or video appointment vs only in-person, pre-COVID and COVID	Telephone/video: 37,660 (37.0) vs 490,199 (43.4)<br>In-person: 64,239 (63.0) vs 638,586 (56.6)	N/A	N/A
		Proportion of participants who had at least one telephone or video appointment vs	Telephone/video: 171 (0.2) vs 5450 (0.6)	N/A	N/A

© 2025 Wennberg E et al. *JAMA Network Open*.

		only in-person, pre-COVID only	In-person: 76,780 (99.8) vs 846,661 (99.4)		
		Proportion of participants who had at least one telephone or video appointment vs only in-person, COVID only	Telephone/video: 37,598 (57.2) vs 488,678 (61.9)<br><br>In-person: 28,112 (42.8) vs 301,230 (38.1)	N/A	N/A
Jallow 2023 (specialist – dermatology)[3]	Non-English speaking or unknown vs English speaking, using electronic medical record	Proportion of visits that were video vs in-person, quarantine period (April to June 2020)	Video: 245 (62.5) vs 1272 (60.9)<br><br>In-person: 147 (37.5) vs 818 (39.1)<br><br>p<0.0001	N/A	N/A
		Proportion of visits that were video vs in-person, post-quarantine period (January to March 2021)	Video: 127 (13.5) vs 360 (9.8)<br><br>In-person: 814 (86.5) vs 3296 (90.2)<br><br>p<0.0001	N/A	N/A
Qian 2022 (specialist – oncology)	Spanish vs English as preferred language in electronic medical record	Proportion of visits that were telephone or video vs in-person	NR	Multivariable mixed effects logistic regression, OR [95% CI] (sex, age at visit, race and ethnicity, marital status, cancer site, median household income, insurance)	0.70 (0.58-0.85), p=0.0004
	Language other than Spanish or English vs English as preferred	Proportion of visits that were telephone or video vs in-person	NR	Multivariable mixed effects logistic regression, OR [95% CI] (sex, age at visit, race and ethnicity, marital	0.75 (0.61-0.93), p=0.008

© 2025 Wennberg E et al. *JAMA Network Open*.

	language in electronic medical record			status, cancer site, median household income, insurance)	
Liang 2022 (specialist – gynecologic oncology)	First language other than English and required interpreter services vs English as first language	Proportion of visits that were telehealth vs in-person	Telehealth: 88 (11.0) vs 3151 (19.6)<br>In-person: 714 (89.0) vs 12,968 (80.4), p<0.01	N/A	N/A
Hundal 2022 (specialist – hematology-oncology)	Use of Spanish vs English language survey	Proportion of participants who self-reported any video or telephone use vs in-person care alone	Telephone/video: 21 (38.2) vs 37 (29.6)<br>In-person: 34 (61.8) vs 88 (70.4)<br>p=0.02	N/A	N/A
	Spanish or other vs English as self-reported primary language spoken at home	Proportion of participants who self-reported any video or telephone use vs in-person care alone	Telephone/video: 24 (37.5) vs 34 (29.3)<br>In-person: 40 (62.5) vs 82 (70.7)	N/A	N/A
	Spanish vs English as self-reported primary language spoken at home	Proportion of participants who self-reported any video or telephone use vs in-person care alone	Telephone/video: 22 (36.1) vs 34 (29.3)<br>In-person: 39 (64.0) vs 82 (70.7)	N/A	N/A
	Language other than Spanish vs English as self-reported primary	Proportion of participants who self-reported any video or telephone use vs in-person care alone	Telephone/video: 2 (66.7) vs 34 (29.3)<br>In-person: 1 (33.3) vs 82 (70.7)	N/A	N/A

© 2025 Wennberg E et al. *JAMA Network Open*.

	language spoken at home				
Neeman 2022 (specialist – hematology-oncology)	Primary language other than English and need for interpreter vs no need for interpreter or unmarked field in electronic health record	Proportion of encounters that were telephone/video vs in-person	Video/telephone: 2845 (86.0) vs 80,390 (89.0)<br>In-person: 462 (14.0) vs 9943 (11.0), p<0.01	N/A	N/A
Chen 2022b (specialist – ophthalmology)	Language other than English vs English as primary language in electronic health record	Proportion of participants with at least 1 video or telephone visit vs in-person alone	Telephone/video: 66 (13.5) vs 1022 (22.5)<br>In-person: 424 (86.5) vs 3511 (77.5)	Bivariable logistic regression, OR [95% CI] (ophthalmic subspecialty)	0.56 (0.42-0.74), p<0.001
				Multivariable logistic regression with Bonferroni correction, OR [95% CI] (age, sex, ethnicity or race, insurance status, household median income quartile, educational attainment quartile, ophthalmic subspecialty)	0.71 (0.50-0.98), p=0.04
Kim 2022a (specialist – physical therapy)	Language other than English vs English as primary language in electronic medical record	Proportion of visits that were telephone or video vs in-person	Telephone/video: 17 (1.5) vs 1999 (3.3)<br>In-person: 1131 (98.5) vs 58,510 (96.7)<br>p=0.001	N/A	N/A
Silverstein 2022 (specialist – minimally invasive gynecologic surgery)	Language other than English vs English as primary language in	Proportion of participants who received a video vs an in-person appointment following referral	Video: 15 (42.9) vs 457 (65.3)	N/A	N/A

© 2025 Wennberg E et al. *JAMA Network Open*.

	electronic medical record		In-person: 20 (57.1) vs 243 (34.7)<br><br>p<0.01		
Kim 2022b (specialist – otolaryngology head and neck surgery)	Language other than English vs English as primary language in medical records	Proportion of participants who completed a telephone vs in-person visit	Telephone: 111 (13.2) vs 143 (15.7)<br><br>In-person: 731 (86.8) vs 767 (84.3)	N/A	N/A
Javier-DesLoges 2022 (specialist – urology)	Spanish vs Cantonese vs other vs English as primary language in medical records	Proportion of participants who completed a telephone vs in-person visit	Telephone: 65 (14.7) vs 23 (11.0) vs 23 (12.0) vs 143 (15.7)<br><br>In-person: 377 (85.3) vs 186 (89.0) vs 168 (88.0) vs 767 (84.3), p=0.26	N/A	N/A
	Spanish vs English as preferred language in medical records	Proportion of visits that were telephone or video vs in-person	Telephone/video: 107 (30.7) vs 1410 (37.7)<br><br>In-person: 242 (69.3) vs 2327 (62.3)	Multivariable logistic regression, OR [95% CI] (race/ethnicity, age, sex, insurance, reason for visit)	0.89 (0.65-1.23), p=0.51
	Language other than Spanish or English vs English as preferred language in medical records	Proportion of visits that were telephone or video vs in-person	Telephone/video: 50 (33.8) vs 1410 (37.7)<br><br>In-person: 98 (66.2) vs 2327 (62.3)	Multivariable logistic regression, OR [95% CI] (race/ethnicity, age, sex, insurance, reason for visit)	0.92 (0.63-1.35), p=0.69

© 2025 Wennberg E et al. *JAMA Network Open*.

Odukoya 2022 (specialist – urology)	Need for interpreter vs no need for interpreter documented in electronic health records	Proportion of participants with any video or telephone visit vs in-person alone	Video/telephone: 33 (60.0) vs 4678 (60.3)<br><br>In-person: 22 (40.0) vs 3079 (39.7)	N/A	N/A

CI = confidence interval; NR = not reported; OR = odds ratio; SE = standard error
Bolded results indicate that the exposure and comparator group or the outcome and the reference as reported in the included study were inverted compared to in our meta-analyses; these results appear as reported in this table but were inverted for the meta-analysis.
[1] Only raw proportions were reported; we estimated raw numbers using these.
[2] Unadjusted odds ratios were reported that were different from those calculated directly from the raw data. We used only the raw data for the meta-analysis and did not extract the reported unadjusted and adjusted odds ratios for our synthesis.
[3] We combined the quarantine and post-quarantine periods for the meta-analysis.

© 2025 Wennberg E et al. *JAMA Network Open*.

eTable 15. Summary of results across studies (n=11) of adult patients facing and not facing language barriers that examined use of video versus telephone visits

Study	Exposure, comparator	Outcome	Raw proportions (n, %)	Model, measure (covariates)	Inferential value
Chen 2022a (primary/ specialist)	Need for vs no need for interpreter documented in electronic health record	Proportion of participants who received any video vs only telephone care	Video: 512 (34.5) vs 56,227 (54.9)<br>Telephone: 946 (65.5) vs 46,254 (45.1)	Multivariable logistic regression, average marginal effect (%) [95% CI] (age, sex, race, ethnicity, primary insurer, rural residence by zip code, neighborhood income quartile, neighborhood broadband access)	-19.3 (-21.8 to -14.4)
Eberly 2020 (primary/ specialist)	Language other than English vs English as preferred language in electronic medical record	Proportion of participants who attended a video vs telephone primary or specialist visit	Video: 748 (41.0) vs 35,040 (45.7)<br>Telephone: 1,077 (59.0) vs 41,618 (54.3)<br>p<0.001	Multivariable logistic regression, OR [95% CI] (age, sex, race/ethnicity, insurance payor, median household income, Charlson Comorbidity Index score)	0.85 (0.76-0.95)
		Proportion of participants who attended a video vs telephone primary care visit	Video: 505 (45.2) vs 17,692 (43.1)<br>Telephone: 613 (54.8) vs 23,400 (56.9)<br>p=0.16	Multivariable logistic regression, OR [95% CI] (age, sex, race/ethnicity, insurance payor, median household income, Charlson Comorbidity Index score)	1.05 (0.91-1.22), p=0.48
		Proportion of participants who attended a video vs telephone specialist care visit	Video: 243 (34.4) vs 17,348 (48.8)<br>Telephone: 464 (65.6) vs 18,218 (51.2)<br>p<0.001	Multivariable logistic regression, OR [95% CI] (age, sex, race/ethnicity, insurance payor, median household income, Charlson Comorbidity Index score)	0.63 (0.53-0.75),<br>p<0.001

© 2025 Wennberg E et al. *JAMA Network Open*.

		Proportion of participants who attended a video vs telephone cardiology clinic visit	Video: 103 (42.0) vs 7165 (57.2)<br>Telephone: 142 (58.0) vs 5353 (42.8)<br>p<0.001	Multivariable logistic regression, OR [95% CI] (age, sex, race/ethnicity, insurance payor, median household income, Charlson Comorbidity Index score)	0.80 (0.60-1.07), p≥0.05
		Proportion of participants who attended a video vs telephone pulmonology clinic visit	Video: 16 (23.2) vs 1377 (41.8)<br>Telephone: 53 (76.8) vs 1918 (58.2)<br>p=0.002	Multivariable logistic regression, OR [95% CI] (age, sex, race/ethnicity, insurance payor, median household income, Charlson Comorbidity Index score)	0.56 (0.29-1.05), p≥0.05
		Proportion of participants who attended a video vs telephone nephrology clinic visit	Video: 12 (30.0) vs 664 (42.3)<br>Telephone: 28 (70.0) vs 907 (57.7)<br>p=0.12	Multivariable logistic regression, OR [95% CI] (age, sex, race/ethnicity, insurance payor, median household income, Charlson Comorbidity Index score)	0.51 (0.24-1.09), p≥0.05
		Proportion of participants who attended a video vs telephone endocrinology clinic visit	Video: 38 (51.4) vs 2036 (54.3)<br>Telephone: 36 (48.6) vs 1716 (45.7)<br>p=0.62	Multivariable logistic regression, OR [95% CI] (age, sex, race/ethnicity, insurance payor, median household income, Charlson Comorbidity Index score)	0.92 (0.55-1.54), p≥0.05
		Proportion of participants who attended a video vs telephone infectious disease clinic visit	Video: 3 (27.3) vs 207 (32.2)<br>Telephone: 8 (72.7) vs 436 (67.8)	Multivariable logistic regression, OR [95% CI] (age, sex, race/ethnicity, insurance payor, median household income, Charlson Comorbidity Index score)	0.81 (0.18-3.56), p≥0.05

© 2025 Wennberg E et al. *JAMA Network Open*.

			p=0.73		
		Proportion of participants who attended a video vs telephone gastroenterology clinic visit	Video: 32 (32.6) vs 2340 (50.3)<br>Telephone: 66 (67.4) vs 2312 (49.7)<br>p=0.001	Multivariable logistic regression, OR [95% CI] (age, sex, race/ethnicity, insurance payor, median household income, Charlson Comorbidity Index score)	0.56 (0.35-0.90), p<0.05
		Proportion of participants who attended a video vs telephone rheumatology clinic visit	Video: 8 (14.0) vs 931 (39.3)<br>Telephone: 49 (86.0) vs 1437 (60.7)<br>p<0.001	Multivariable logistic regression, OR [95% CI] (age, sex, race/ethnicity, insurance payor, median household income, Charlson Comorbidity Index score)	0.27 (0.12-0.59), p<0.05
		Proportion of participants who attended a video vs telephone hematology-oncology clinic visit	Video: 31 (27.4) vs 2628 (38.8)<br>Telephone: 82 (72.6) vs 4139 (61.2)<br>p=0.01	Multivariable logistic regression, OR [95% CI] (age, sex, race/ethnicity, insurance payor, median household income, Charlson Comorbidity Index score)	0.51 (0.32-0.82), p<0.05
Khatana 2022 (primary/ specialist)	**English vs language other than English** as primary language in electronic medical records	Proportion of participants with any video vs telephone-only virtual encounters	NR	Multivariable mixed effects logistic regression, OR [95% CI] (age; sex; race/ethnicity; comorbidities; proportion of rural residents living in the 3-digit ZIP code of residence; socioeconomic, household composition and disability, minority status and language index, and housing and transportation components of the social vulnerability index for the 3-digit ZIP code of	**2.05 (1.83-2.31), p<0.001**

© 2025 Wennberg E et al. *JAMA Network Open*.

				residence; random intercepts for medical provider)	
Le 2022 (primary/ specialist)	Ever vs never use of interpreter services in administrative billing data	Proportion of participants with any video vs only telephone or secure messaging vs secure messaging alone virtual encounters	Video: 1908 (54.8) vs 9140 (53.0)<br>Telephone: 1505 (43.2) vs 7999 (46.4)<br>Secure messaging: 68 (2.0) vs 96 (0.6)	N/A	N/A
Zachrison 2023 (primary/ specialist)[1]	Language other than English vs English as preferred language in electronic health record	Proportion of participants with any video vs telephone-only virtual visits	Video: 22,437 (59.7) vs 347,234 (71.1)<br>Telephone: 15,161 (40.3) vs 141,444 (28.9)	N/A	N/A
Hsueh 2021 (primary)	Need for interpreter vs no need for interpreter documented in electronic health records	Proportion of telemedicine visits self-scheduled as video vs telephone	Video: 7765 (34.5) vs 371,237 (39.8)<br>Telephone: 14,711 (65.5) vs 561,639 (60.2)	Multivariable logistic regression, OR [95% CI] (race/ethnicity, age, gender, neighborhood SES, neighborhood internet, mobile portal use, prior video visit experience in past 12 months, visit with own primary care provider, time, medical service area, ICD-10 grouping)	0.77 (0.74-0.80), p<0.001
				Multivariable logistic regression, adjusted video visit frequencies (%) (race/ethnicity, age, gender, neighborhood SES, neighborhood internet, mobile portal use, prior video visit experience in past 12 months, visit with own primary care	34.7 vs 39.8, p<0.001

© 2025 Wennberg E et al. *JAMA Network Open*.

				provider, time, medical service area, ICD-10 grouping)	
		Proportion of telemedicine visits scheduled as video vs telephone, patients without prior video visit experience subgroup	NR	Multivariable logistic regression, adjusted video visit frequencies (%) (need for interpreter*prior video visit experience in past 12 months, race/ethnicity, age, gender, neighborhood SES, neighborhood internet, mobile portal use, visit with own primary care provider, time, medical service area, ICD-10 grouping)	28.9 vs 35.9, p<0.001
		Proportion of telemedicine visits scheduled as video vs telephone, patients with prior video visit experience subgroup	NR	Multivariable logistic regression, adjusted video visit frequencies (%) (need for interpreter*prior video visit experience in past 12 months, race/ethnicity, age, gender, neighborhood SES, neighborhood internet, mobile portal use, visit with own primary care provider, time, medical service area, ICD-10 grouping)	47.2 vs 49.1, p=0.09
Rowe 2021 (specialist – cardiology)	Language other than English vs English as preferred language in electronic medical record	Proportion of participants who used video vs telephone for their initial appointment in the study period	Video: 33 (17.1) vs 294 (22.2)<br>Telephone: 160 (82.9) vs 1028 (77.8), p=0.04	N/A	N/A
Liu 2021 (specialist – geriatric medicine)	**English vs non-English language** as language of assessment	Proportion of participants who received a video vs telephone virtual assessment	Video: 89 (31.0) vs 14 (32.6)	Univariable logistic regression, OR [95% CI]	**0.93 (0.47-1.85)**
				Multivariable logistic regression, OR [95% CI] (age, sex, clinical frailty scale, education, immigration history,	**1.24 (0.54-2.86)**

© 2025 Wennberg E et al. *JAMA Network Open*.

			Telephone: 198 (69.0) vs 29 (67.4), p=0.84	history of cognitive impairment, caregiver involvement at assessment, ability to use a computer)	
				Multivariable logistic regression, risk difference [95% CI] (age, sex, clinical frailty scale, education, immigration history, history of cognitive impairment, caregiver involvement at assessment, ability to use a computer)	**0.04 (-0.11 to 0.18)**
Neeman 2022 (specialist – hematology-oncology)	Primary language other than English and need for interpreter vs no need for interpreter or unmarked field in electronic health record	Proportion of virtual encounters that were video vs telephone	Video: 810 (28.5) vs 30,433 (37.8)<br>Telephone: 2035 (71.5) vs 49,957 (62.1), p<0.01	N/A	N/A
Chen 2022b (specialist – ophthalmology)	Language other than English vs English as primary language in electronic health record	Proportion of telemedicine recipients with **telephone visits alone vs any video visit**	Telephone: 49 (74.2) vs 593 (58.0)<br>Video: 17 (25.8) vs 429 (42.0)	Bivariable logistic regression, OR [95% CI] (ophthalmic subspecialty)	**2.30 (1.34-4.10), p=0.003**
				Multivariable logistic regression with Bonferroni correction, OR [95% CI] (age, sex, ethnicity or race, insurance status, household median income quartile, educational attainment quartile, ophthalmic subspecialty)	**2.36 (1.19-4.87), p=0.02**
Odukoya 2022 (specialist – urology)	Need for interpreter vs no need for interpreter	Proportion of participants with any video vs only telephone visits	Any video: 16 (48.5) vs 2639 (56.4)	Multivariable logistic regression, average marginal effect (%) [95% CI] (age, sex, race, ethnicity, primary insurer, rural residence, households	-4.5 (-5.0 to -3.1)

© 2025 Wennberg E et al. *JAMA Network Open*.

	documented in electronic health records		Telephone only: 17 (51.5) vs 2039 (43.6)	below poverty, households with broadband access)	

CI = confidence interval; NR = not reported; OR = odds ratio
Bolded results indicate that the exposure and comparator group or the outcome and the reference as reported in the included study were inverted compared to in our meta-analyses; these results appear as reported in this table but were inverted for the meta-analysis.
[1]Unadjusted odds ratios were reported that were different from those calculated directly from the raw data. We used only the raw data for the meta-analysis and did not extract the reported unadjusted and adjusted odds ratios for our synthesis.

© 2025 Wennberg E et al. *JAMA Network Open*.

eTable 16. Summary of results across studies (n=7) of adult patients facing and not facing language barriers that examined non-completion versus completion of scheduled virtual care visits

Study	Exposure, comparator	Outcome	Raw proportions (n, %)	Model, measure (covariates)	Inferential value
Eberly 2020 (primary/ specialist)	Language other than English vs English as preferred language in electronic medical record	Proportion who **completed vs who did not complete** a scheduled telephone or video primary/specialist care visit	Completed: 1905 (48.9) vs 78,819 (54.7)<br>Did not complete: 1990 (51.1) vs 65,280 (45.3)<br>p<0.001	Multivariable logistic regression, OR [95% CI] (age, sex, race/ethnicity, payor class, median household income category, Charlson comorbidities score)	**0.84 (0.78-0.90)**
		Proportion who **completed vs who did not complete** a scheduled telephone or video primary care visit	Completed: 1173 (56.5) vs 41,898 (56.8)<br>Did not complete: 903 (43.5) vs 31,867 (43.2)<br>p=0.79	Multivariable logistic regression, OR [95% CI] (age, sex, race/ethnicity, payor class, median household income category, Charlson comorbidities score)	**0.98 (0.88-1.08), p=0.64**
		Proportion who **completed vs who did not complete** a scheduled telephone or video specialist care visit	Completed: 732 (40.2) vs 36,921 (52.5)<br>Did not complete: 1087 (59.8) vs 33,413 (47.5)<br>p<0.001	Multivariable logistic regression, OR [95% CI] (age, sex, race/ethnicity, payor class, median household income category, Charlson comorbidities score)	**0.69 (0.62-0.77), p<0.001**
		Proportion who **completed vs who did not complete** a scheduled telephone or video cardiology clinic visit	Completed: 258 (40.1) vs 12771 (50.7)<br>Did not complete: 385 (59.9) vs 12439 (49.3)<br>p<0.001	Multivariable logistic regression, OR [95% CI] (age, sex, race/ethnicity, insurance payor, median household income, Charlson Comorbidity Index score)	**0.68 (0.57-0.82), p<0.05**

© 2025 Wennberg E et al. *JAMA Network Open*.

		Proportion who **completed vs who did not complete** a scheduled telephone or video pulmonology clinic visit	Completed: 73 (41.7) vs 3469 (54.6)<br><br>Did not complete: 102 (58.3) vs 2883 (45.4)<br><br>p=0.001		**0.80 (0.56-1.13), p≥0.05**
		Proportion who **completed vs who did not complete** a scheduled telephone or video nephrology clinic visit	Completed: 40 (48.8) vs 1650 (64.7)<br><br>Did not complete: 42 (51.2) vs 901 (35.3)<br><br>p=0.003		**0.73 (0.44-1.20), p≥0.05**
		Proportion who **completed vs who did not complete** a scheduled telephone or video endocrinology clinic visit	Completed: 76 (38.8) vs 3945 (54.8)<br><br>Did not complete: 120 (61.2) vs 3259 (45.2)<br><br>p<0.001		**0.61 (0.44-0.84), p<0.05**
		Proportion who **completed vs who did not complete** a scheduled telephone or video infectious disease clinic visit	Completed: 13 (34.2) vs 700 (49.8)<br><br>Did not complete: 25 (65.8) vs 706 (50.2)<br><br>p=0.06		**0.45 (0.21-0.96), p<0.05**
		Proportion who **completed vs who did not complete** a scheduled telephone or video gastroenterology clinic visit	Completed: 98 (35.6) vs 4827 (50.3)<br><br>Did not complete: 177 (64.4) vs 4770 (49.7)<br><br>p<0.001		**0.61 (0.46-0.80), p<0.05**

© 2025 Wennberg E et al. *JAMA Network Open*.

		Proportion who **completed vs who did not complete** a scheduled telephone or video rheumatology clinic visit	Completed: 59 (63.4) vs 2499 (63.4)<br><br>Did not complete: 34 (36.6) vs 1446 (36.6)<br><br>p=0.99		**1.29 (0.78-2.11), p≥0.05**
		Proportion who **completed vs who did not complete** a scheduled telephone or video hematology-oncology clinic visit	Completed: 115 (36.3) vs 7060 (50.2)<br><br>Did not complete: 202 (63.7) vs 7009 (49.8)<br><br>p<0.001		**0.60 (0.47-0.78), p<0.05**
Gmunder 2021 (primary/ specialist)	Language other than English vs English as preferred language in electronic health record	Proportion of **completed vs non-completed** scheduled video visits	Completed: 60,868 (59.7) vs 168,523 (64.9)<br><br>Non-completed: 41,082 (40.3) vs 91,191 (35.1)	N/A	N/A
	Spanish vs English as preferred language in electronic health record	Proportion of **completed vs non-completed** scheduled video visits	Completed: 58,732 (59.8) vs 168,523 (64.9)<br><br>Non-completed: 39,462 (40.2) vs 91,191 (35.1)	Mixed effects multivariable logistic regression, OR (insurance, race, age, ethnicity, sex, religion, weighted average income, pre-visit phone/text confirmation status, MyUHealthChart status, provider specialty, new to provider, new to UHealth, random effect for clinical site)	**0.91, p<0.001**
	Language other than Spanish/English vs English as preferred language in	Proportion of **completed vs non-completed** scheduled video visits	Completed: 2136 (56.9) vs 168,523 (64.9)<br><br>Non-completed: 1620 (43.1) vs 91,191 (35.1)	Mixed effects multivariable logistic regression, OR (insurance, race, age, ethnicity, sex, religion, weighted average income, pre-visit phone/text confirmation status, MyUHealthChart status, provider	**0.89, p<0.01**

© 2025 Wennberg E et al. *JAMA Network Open*.

	electronic health record			specialty, new to provider, new to UHealth, random effect for clinical site)	
Chen 2022c (primary)	Spanish vs English as primary language in electronic health record	Proportion of non-attended vs attended scheduled telephone visits, telehealth transition period (March-June 2020)	NR	Hierarchical multiple logistic regression, OR [95% CI] (age, sex, race/ethnicity, insurance, Elixhauser comorbidity count; random effects for patients and clinics)	0.88 (0.81-0.95)
		Proportion of non-attended vs attended scheduled telephone visits, elective telehealth period (July 2020-August 2021)	NR	Hierarchical multiple logistic regression, OR [95% CI] (age, sex, race/ethnicity, insurance, Elixhauser comorbidity count; random effects for patients and clinics)	0.99 (0.93-1.05)
		Proportion of non-attended vs attended scheduled video visits, elective telehealth period (July 2020-August 2021)	NR	Hierarchical multiple logistic regression, OR [95% CI] (age, sex, race/ethnicity, insurance, Elixhauser comorbidity count; random effects for patients and clinics)	1.25 (0.99-1.57)
	Language other than Spanish or English vs English as primary language in electronic health record	Proportion of non-attended vs attended scheduled telephone visits, telehealth transition period (March-June 2020)	NR	Hierarchical multiple logistic regression, OR [95% CI] (age, sex, race/ethnicity, insurance, Elixhauser comorbidity count; random effects for patients and clinics)	0.97 (0.92-1.02)
		Proportion of non-attended vs attended scheduled telephone visits, elective telehealth period (July 2020-August 2021)	NR	Hierarchical multiple logistic regression, OR [95% CI] (age, sex, race/ethnicity, insurance, Elixhauser comorbidity count; random effects for patients and clinics)	1.05 (1.00-1.10)

© 2025 Wennberg E et al. *JAMA Network Open*.

		Proportion of non-attended vs attended scheduled video visits, elective telehealth period (July 2020-August 2021)	NR	Hierarchical multiple logistic regression, OR [95% CI] (age, sex, race/ethnicity, insurance, Elixhauser comorbidity count; random effects for patients and clinics)	1.93 (1.62-2.31)
Pitaro 2022 (specialist – orthopedics)	Language other than English vs English as primary language spoken	Proportion of participants who did not attend vs who attended scheduled virtual total joint arthroplasty education classes	Did not attend: 22 (14.4) vs 370 (33.0)<br>Attended: 131 (85.6) vs 752 (67.0)<br>Standardized difference=0.308	N/A	N/A
Kim 2022b (specialist – otolaryngology head and neck surgery)	Language other than English vs English as primary language in medical records	Proportion of participants who missed vs who completed a telephone visit	Missed: 19 (14.6) vs 24 (14.4)<br>Completed: 111 (85.4) vs 143 (85.6)	N/A	N/A
	Spanish vs Cantonese vs other vs English as primary language in medical records	Proportion of participants who missed vs who completed a telephone visit	Missed: 10 (13.3) vs 5 (17.9) vs 4 (14.8) vs 24 (14.4)<br>Completed: 65 (86.7) vs 23 (82.1) vs 23 (85.2) vs 143 (85.6), p=0.95	N/A	N/A
Abou Ali 2023 (specialist – vascular surgery)	Language other than English vs English as spoken	Proportion of **completed vs non-completed** scheduled	NR	Multivariable logistic regression, OR [95% CI] (age, sex, race, ethnicity, visit type)	**1.24 (0.87-1.78), p=0.24**

© 2025 Wennberg E et al. *JAMA Network Open*.

	language in electronic medical record	telephone and video visits			
Bell 2022 (specialist – urology)	Language other than English vs English as primary language in electronic medical record	Proportion of non-attended vs attended scheduled telephone visits	Non-attended: 34 (26.2) vs 60 (31.2)<br><br>Attended: 96 (73.8) vs 132 (68.8)	N/A	N/A
	By specific primary language		Non-attended: Arabic: 0 (0.0); Cantonese: 6 (20.7); Mandarin: 1 (20.0); Russian: 0 (0.0); Spanish: 21 (30.0); Tagalog: 0 (0.0); Other: 6 (46.2); English: 60 (31.2)<br><br>Attended: Arabic: 2 (100); Cantonese: 23 (79.3); Mandarin: 4 (80.0); Russian: 7 (100.0); Spanish: 49 (70.0); Tagalog: 4 (100.0); Other: 7 (53.8); English: 132 (68.8)<br><br>p=0.27	N/A	N/A

CI = confidence interval; NR = not reported; OR = odds ratio
Bolded results indicate that the exposure and comparator group or the outcome and the reference as reported in the included study were inverted compared to in our meta-analyses; these results appear as reported in this table but were inverted for the meta-analysis.

© 2025 Wennberg E et al. *JAMA Network Open*.

eTable 17. Summary of results across studies (n=6) of adult patients facing and not facing language barriers that examined ever versus never use of virtual care

Study	Exposure, comparator	Outcome	Raw proportions (n, %)	Model, measure (covariates)	Inferential value
Andersen 2021 (primary/ specialist)	Self-reported English speaking ability of not well vs very well or well	Proportion of participants who self-reported use vs no use of video visits	Used: 5 (38.5) vs 13 (13.5)<br>Not used: 8 (61.5) vs 83 (86.5)	N/A	N/A
	Self-reported English speaking ability of not well vs well vs very well	Proportion of participants who self-reported use vs no use of video visits	Used: 5 (38.5) vs 8 (15.7) vs 5 (11.1)<br>Not used: 8 (61.5) vs 43 (84.3) vs 40 (88.9) p=0.075	N/A	N/A
Chang 2022 (primary/ specialist)	Self-reported language other than English spoken at home vs English spoken at home	Proportion of participants who self-reported use of one or more vs no telephone or video visits	NR	Weighted multivariable logistic regression, weighted OR [95% CI] (age, sex, race/ethnicity, metropolitan residence, annual income, Medicare-Medicaid dual eligibility, had equipment to access telehealth)	0.86 (0.64-1.15), p=0.3
El-Toukhy 2020 (primary/ specialist)	Self-reported English ability of well, not well, or not at all vs very well	Proportion of participants who self-reported use vs non-use of secure messaging with health care provider and staff via patient portal	NR	Multivariable logistic regression, OR [95% CI] (gender, age, race/ethnicity, education, marital status, health insurance, regular provider, general health, census region, rural/urban)	0.8 (0.4-1.3)
Le 2022 (primary/ specialist)	Ever vs never use of interpreter services in administrative billing data	Proportion of participants with any vs no telephone, video, or secure messaging encounter	Any virtual: 3481 (30.4) vs 17,235 (22.5)<br>No virtual: 7970 (69.6) vs 59,511 (77.5)	N/A	N/A

© 2025 Wennberg E et al. *JAMA Network Open*.

Rodriguez 2021 (primary/ specialist)	"Limited English proficiency" (English speaking ability of not well or not at all, survey response) vs "English proficiency"	Proportion who self-reported receipt vs no receipt of care from a doctor or health professional through a video or telephone conversation rather than an office visit	Used: 366 (4.8) vs 9381 (12.3)<br>Not used: 7697 (95.2) vs 66,975 (87.7)<br>p<0.001	Weighted univariable logistic regression, OR	0.36, p<0.001
				Weighted multivariable logistic regression, OR (age, sex, percent of federal poverty level, race/ethnicity, health status, insurance, source of care, location)	0.56, p<0.001
		Proportion who self-reported receipt vs no receipt of care from a doctor or health professional through a video or telephone conversation rather than an office visit, 2015-2016 subgroup (with internet use variable)	NR	Weighted univariable logistic regression, OR	0.35, p<0.001
				Weighted multivariable logistic regression, OR (age, sex, percent of federal poverty level, race/ethnicity, health status, insurance, source of care, location, internet use)	0.60, p<0.05
Ramsey 2022 (specialist – ophthalmology)	Language other than English vs English as primary language spoken recorded in electronic medical record	Proportion of participants who used vs did not use a telephone or video visit in study period, limited to first visit in study period	Used telephone/video: 100 (22.7) vs 1639 (21.3)<br>Did not use: 340 (77.3) vs 6068 (78.7)<br>p=0.467	N/A	N/A

CI = confidence interval; NR = not reported; OR = odds ratio
Bolded results indicate that the exposure and comparator group or the outcome and the reference as reported in the included study were inverted compared to in our meta-analyses; these results appear as reported in this table but were inverted for the meta-analysis.

eTable 18. Summary of results across studies (n=4) of adult patients facing and not facing language barriers that examined other use of virtual care outcomes

© 2025 Wennberg E et al. *JAMA Network Open*.

Study	Exposure, comparator	Outcome	Raw proportions (n, %)	Model, measure (covariates)	Inferential value
Chen 2022b (specialist – ophthalmology)	Language other than English vs English as primary language in electronic health record	Proportion of participants with any video visit vs telephone alone or in-person alone	Video: 17 (3.5) vs 429 (9.5)<br>Telephone or in-person: 473 (96.5) vs 4104 (90.5)	Bivariable logistic regression, OR [95% CI] (ophthalmic subspecialty)	0.34 (0.21-0.54), p<0.001
				Multivariable logistic regression with Bonferroni correction, OR [95% CI] (age, sex, ethnicity or race, insurance status, household median income quartile, educational attainment quartile, ophthalmic subspecialty)	0.49 (0.28-0.82), p=0.01
Neeman 2022 (specialist – hematology-oncology)	Primary language other than English and need for interpreter vs no need for interpreter or unmarked field in electronic health record	Median number (n, IQR) of secure messages sent and received per patient	0 (0-3) vs 2 (0-8), p<0.01	N/A	N/A
Tong 2022 (specialist – oncology)	Need for vs no need for interpreter documented in electronic medical record	Proportion of participants who used any video vs in-person alone or in-person and telephone/text message	Video: 104 vs 14,046<br>In-person: 483 vs 31,819	Univariable logistic regression, OR [95% CI]	0.49 (0.39-0.60), p<0.001
		Proportion of participants who used any video vs only telephone/text message and/or in-person	NR	Multivariable logistic regression, OR [95% CI] (age, sex, race, ethnicity, employment status, insurance status, area deprivation index, rurality)	0.40 (0.30-0.52), p<0.001

© 2025 Wennberg E et al. *JAMA Network Open*.

		Proportion of participants who used any telephone/text message vs only video and/or in-person	NR	Multivariable logistic regression, OR [95% CI] (age, sex, race, ethnicity, employment status, insurance status, area deprivation index, rurality)	1.14 (0.86-1.52, p=0.365
		Proportion of participants who used any in-person vs only video and/or telephone/text message	NR	Multivariable logistic regression, OR [95% CI] (age, sex, race, ethnicity, employment status, insurance status, area deprivation index, rurality)	1.64 (1.27-2.12), p<0.001
Wood 2021 (specialist – HIV care)	Language other than English vs English as primary language	Proportion of participants who completed any video visit vs only phone and/or in-person visits	Video: 65 (21.5) vs 672 (34.7)<br><br>Phone and/or in-person: 237 (78.5) vs 1266 (65.3)<br><br>p=0.1315	N/A	NR
	Spanish vs English as primary language	Proportion of participants who completed any video visit vs only phone and/or in-person visits	Video: 33 (27.5) vs 672 (34.7)<br><br>Phone and/or in-person: 87 (72.5) vs 1266 (65.3)	N/A	NR
	Language other than Spanish or English vs English as primary language	Proportion of participants who completed any video visit vs only phone and/or in-person visits	Video: 32 (17.6) vs 672 (34.7)<br><br>Phone and/or in-person: 150	N/A	NR

© 2025 Wennberg E et al. *JAMA Network Open*.

			(82.4) vs 1266 (65.3)		

CI= confidence interval; IQR = interquartile range; NR = not reported; OR = odds ratio
Bolded results indicate that the exposure and comparator group or the outcome and the reference as reported in the included study were inverted compared to in our meta-analyses; these results appear as reported in this table but were inverted for the meta-analysis.

© 2025 Wennberg E et al. *JAMA Network Open*.

eTable 19. Summary of results across studies (n=3) of caregivers of pediatric patients facing and not facing language barriers that examined use of virtual versus in-person care

Study	Exposure, comparator	Outcome	Raw proportions (n, %)	Model, measure (covariates)	Inferential value
Cahan 2022 (specialist – 6 pediatric subspecialties)	Family request for vs no family request for interpreter support in clinical encounter data	Proportion of visits completed as telephone or video vs in-person	NR	Univariable logistic regression, OR [95% CI]	0.56 (0.51-0.61)
				Multivariable logistic regression, OR [95% CI] (new vs established patient, age, sex, race/ethnicity, insurance type, distance from clinic, income status, broadband availability, subspecialty)	0.68 (0.60-0.78)
Dai 2021 (specialist - otolaryngology)	Primary language other than English and required or requested an interpreter vs English primary language and/or no request/requirement for interpreter in electronic medical record	Proportion of **ED visits or primary care provider visits vs telephone calls** for follow-up care	ED/primary care provider: 14 (66.7) vs 9 (31.0)<br>Telephone: 7 (33.3) vs 20 (69.0)	N/A	N/A
		Proportion of **ED visits vs telephone calls** for follow-up care	ED: 13 (65.0) vs 4 (16.7)<br>Telephone: 7 (35.0) vs 20 (83.3)	Univariable logistic regression, OR [95% CI]	**9.3 (2.3-38.2)**
		Proportion of **primary care provider visits vs telephone calls** for follow-up care	Primary care provider: 1 (12.5) vs 5 (20.0)<br>Telephone: 7 (87.5) vs 20 (80.0)	Univariable logistic regression, OR [95% CI]	**0.6 (0.06-5.8)**
		Proportion of primary care provider vs ED visits vs telephone calls for follow-up care	Primary care provider: 1 (4.8) vs 5 (17.2)<br>ED: 13 (61.9) vs 4 (13.8)	N/A	N/A

© 2025 Wennberg E et al. *JAMA Network Open*.

			Telephone: 7 (33.3) vs 20 (69.0)<br><br>p=0.002		
		Proportion of **ED visits vs telephone calls** for follow-up care, public insurance subgroup (n=35 visits)	ED: 13 (68.4) vs 3 (21.4)<br><br>Telephone: 6 (31.6) vs 11 (78.6)	Univariable logistic regression, OR [95% CI]	**7.9 (1.6-39.4)**
		Proportion of **primary care provider visits vs telephone calls** for follow-up care, public insurance subgroup (n=35 visits)	Primary care provider: 1 (14.3) vs 1 (8.3)<br><br>Telephone: 6 (85.7) vs 11 (91.7)	Univariable logistic regression, OR [95% CI]	**1.8 (0.1-34.8)**
		Proportion of **ED visits or primary care provider visits vs telephone calls** for follow-up care, public insurance subgroup (n=35 visits)	ED/primary care provider: 14 (70.0) vs 4 (26.7)<br><br>Telephone: 6 (30.0) vs 11 (73.3)	N/A	N/A
		Proportion of primary care provider vs ED visits vs telephone calls for specialist follow-up care, public insurance subgroup (n=35 visits)	Primary care provider: 1 (5.0) vs 1 (6.7)<br><br>ED: 13 (65.0) vs 3 (20.0) Telephone: 6 (30.0) vs 11 (73.3)	N/A	N/A

© 2025 Wennberg E et al. *JAMA Network Open*.

			p=0.04		
Vaughan 2022 (specialist – cardiology)	Language other than English vs English as primary language	Proportion of participants with any telemedicine visit vs in-person only	Telemedicine: 120 (12.2) vs 571 (12.9)<br><br>In-person: 862 (87.8) vs 3857 (87.1)	N/A	N/A
	Spanish vs English as primary language	Proportion of participants with any telemedicine visit vs in-person only	Telemedicine: 112 (12.3) vs 571 (12.9)<br><br>In-person: 802 (87.7) vs 3857 (87.1)	Univariable logistic regression, OR [95% CI]	0.94 (0.76-1.17), p=0.6
	Other/unable to obtain vs English as primary language	Proportion of participants with any telemedicine visit vs in-person only	Telemedicine: 8 (11.8) vs 571 (12.9)<br><br>In-person: 60 (88.2) vs 3857 (87.1)	Univariable logistic regression, OR [95% CI]	0.90 (0.43-1.89), p=0.78

CI = confidence interval; ED = emergency department; NR = not reported; OR = odds ratio
Bolded results indicate that the exposure and comparator group or the outcome and the reference as reported in the included study were inverted compared to in our meta-analyses; these results appear as reported in this table but were inverted for the meta-analysis.

© 2025 Wennberg E et al. *JAMA Network Open*.

eTable 20. Summary of results across studies (n=3) of adult patients facing and not facing language barriers that examined satisfaction with virtual care

Study	Exposure and comparator	Measure	Raw values	Model, measure (covariates)	Inferential value
Choi 2022 (specialist – otolaryngology)	Language other than English vs English as self-reported preferred language	Mean overall scores, Press Ganey survey	NR	Multivariable linear regression, beta coefficient [95% CI] (encounter modality, age, sex, race, education, income, employment, commute to clinic, encounter type, encounter duration, attitudes towards COVID-19)	5.66 (-6.03 to 17.36), p=0.34
		Mean overall scores, Telehealth Satisfaction Questionnaire	NR	Multivariable linear regression, beta coefficient [95% CI] (encounter modality, age, sex, race, education, income, employment, commute to clinic, encounter type, encounter duration, attitudes towards COVID-19)	0.27 (-0.66-1.20), p=0.57
Futterman 2021 (specialist – prenatal care)	Language other than English (100% Spanish) vs English as self-reported primary language	Median overall scores (IQR), Short Assessment of Patient Satisfaction (SAPS)	23.0 (20.0-25.0) vs 22.0 (20.2-26.0)<br>p=0.687	N/A	N/A
Mortezavi 2021 (specialist - rheumatology)	Interpreter needed vs no interpreter needed	Proportion of participants with top 2 box vs neutral vs bottom 2 box scores on “I was satisfied with my telemedicine encounter” (%)	Top 2 box: 58.3 vs 75.2<br>Neutral: 8.3 vs 14.4<br>Bottom 2 box: 33.4 vs 9.9<br>p=0.0011	N/A	N/A

© 2025 Wennberg E et al. *JAMA Network Open*.

		Proportion who responded yes vs no to “I would have preferred an in-person visit instead of a telemedicine visit”	NR<br><br>p=0.09	N/A	N/A

CI = confidence interval; IQR = interquartile range; NR = not reported

© 2025 Wennberg E et al. *JAMA Network Open*.

eTable 21. Summary of results across studies (n=3) of caregivers of pediatric patients facing and not facing language barriers that examined satisfaction with virtual care

Study	Exposure and comparator	Measure	Raw values	Model, measure (covariates)	Inferential value
Hiscock 2022 (primary/specialist)	Language other than English vs English as self-reported main language spoken at child's home	Proportion of participants with top 2 vs bottom 2 box responses on "telehealth was convenient for me and my child"	232 (97.9) vs 2052 (98.0)	N/A	N/A
		Proportion of participants with top 2 vs bottom 2 box responses on "telehealth saved me time"	233 (97.9) vs 2046 (97.9)	N/A	N/A
		Proportion of participants with top 2 vs bottom 2 box responses on "I was comfortable using telehealth technology"	231 (97.9) vs 2057 (98.3)	N/A	N/A
		Proportion of participants with top 2 vs bottom 2 box responses on "joining the telehealth call was easy for me"	237 (99.6) vs 2054 (98.0)	N/A	N/A
		Proportion of participants with top 2 vs bottom 2 box responses on "my child's safety and privacy was maintained"	234 (99.6) vs 2079 (99.9)	N/A	N/A
		Proportion of participants with top 2 vs bottom 2 box responses on "telehealth is	221 (96.1) vs 1966 (95.3)	N/A	N/A

© 2025 Wennberg E et al. *JAMA Network Open*.

		an acceptable way to receive healthcare”			
		Proportion of participants with top 2 vs bottom 2 box responses on “telehealth improves access to health care”	215 (96.0) vs 1978 (96.3)	N/A	N/A
		Proportion of participants with top 2 vs bottom 2 box responses on “I prefer to have the option of telehealth for my child”	202 (89.0) vs 1896 (92.4)	N/A	N/A
		Proportion of participants with top 2 vs bottom 2 box responses on “I prefer in-person over telehealth for my child”	168 (77.8) vs 1188 (61.4)	N/A	N/A
		Proportion of participants with top 2 vs bottom 2 box responses on “telehealth gave the opportunity to ask questions about my child's care”	231 (97.9) vs 2054 (99.0)	N/A	N/A
		Proportion of participants with top 2 vs bottom 2 box responses on “questions and concerns were adequately addressed”	233 (98.7) vs 2065 (99.1)	N/A	N/A
		Proportion of participants with top 2 vs bottom 2 box responses on, “the	194 (83.6) vs 1681 (81.6)	N/A	N/A

© 2025 Wennberg E et al. *JAMA Network Open*.

		telehealth service was as good as in-person"			
Cockrell 2022 (specialist - surgery)	Spanish vs English preferred language of care	Proportion of top box responses (9 or 10 on 0-10 numeric scale; 4 on 4-point Likert scale); 9 questions from Family Experience Survey	NR	Univariable generalized linear regression, incidence rate ratios (95% CI)	Explaining: 1.16 (0.35-2.83)<br><br>Listening: 1.08 (0.33-2.63)<br><br>Questions: 1.33 (0.40-3.25)<br><br>Understanding: 0.84 (0.21-2.27)<br><br>Medical History Knowledge: 0.94 (0.23-2.56)<br><br>Respect: 1.03 (0.31-2.50)<br><br>Time: 0.92 (0.22-2.50)<br><br>Provider Rating: 1.03 (0.72-1.44)<br><br>Office Recommendation: 1.08 (0.75-1.50)
Hanna 2022 (orthopedic surgery)	Primary language other than English vs primary language of English or bilingual including	Mean overall score (SD), Family Experience Survey	3.40 (0.47) vs 3.72 (0.33),<br><br>p=0.017	N/A	N/A
		Mean score on length of time spent with surgeon	3.00 (0.77) vs 3.70 (0.53),	N/A	N/A

© 2025 Wennberg E et al. *JAMA Network Open*.

	English in patient chart	item (SD), Family Experience Survey	p=0.005		
		Mean score on explanation provided by surgeon (SD), Family Experience Survey	3.20 (0.60) vs 3.73 (0.52),<br><br>p=0.016	N/A	N/A

NR = not reported; SD = standard deviation

© 2025 Wennberg E et al. *JAMA Network Open*.

eFigure 1. ROBINS-E risk of bias assessment of included studies (n=41)

Risk of bias domains

Study	D1	D2	D3	D4	D5	D6	D7	Overall
Abou Ali 2023	X	-	+	+	+	+	-	X
Andersen 2021	X	X	-	+	+	+	-	X
Bell 2022	X	X	+	+	+	+	-	X
Cahan 2022	X	+	+	+	X	+	-	X
Chang 2022	X	-	X	+	+	-	-	X
Chen 2021a	X	-	+	+	-	+	-	X
Chen 2021b	X	+	+	+	+	+	-	X
Chen 2022	X	-	+	+	+	+	-	X
Choi 2022	X	+	X	+	X	X	-	!
Cockrell 2022	X	-	X	+	+	-	-	X
Dai 2021	X	+	+	+	+	+	-	X
Eberly 2020	X	-	+	+	+	+	-	X
El-Toukhy 2020	X	-	-	+	+	-	-	X
Futterman 2020	X	-	-	+	+	-	-	X
Gmunder 2021	X	-	+	+	X	+	-	X
Hanna 2022	X	+	X	+	+	-	-	X
Hiscock 2022	X	+	X	+	X	-	-	!
Hsueh 2021	X	-	X	+	+	+	-	X
Hundal 2022	X	-	-	+	X	+	-	X
Jallow 2022	X	X	+	+	X	+	-	!
Javier-DesLoges 2021	X	X	-	+	+	-	-	X
Khatana 2022	-	-	+	+	-	+	-	X
Kim 2022a	X	+	+	+	+	+	-	X
Kim 2022b	X	+	+	+	+	+	-	X
Le 2022	X	+	+	+	+	+	-	X
Liang 2022	X	X	-	+	X	-	-	X
Liu 2021	-	+	+	+	X	+	-	X
Mortezavi 2021	X	+	+	+	+	+	-	X
Neeman 2022	X	-	+	+	X	+	-	X
Odukoya 2022	X	+	+	+	+	+	-	X
Pitaro 2022	X	X	+	+	+	+	-	X
Qian 2022	X	+	-	+	+	+	-	X
Ramsey 2022	X	+	-	+	+	+	-	X
Rodriguez 2021	X	-	-	+	-	+	-	X
Rowe 2021	X	+	+	+	-	+	-	X
Silverstein 2022	X	+	+	+	X	+	-	X
Tong 2022	X	+	+	+	X	+	-	X
Vaughan 2022	X	X	-	+	+	-	-	X
Weber 2020	X	-	+	+	X	+	-	X
Wood 2021	X	X	-	+	+	+	-	X
Zachrison 2021	X	+	+	+	-	+	-	X

Domains:
D1: Bias due to confounding.
D2: Bias arising from measurement of the exposure.
D3: Bias in selection of participants into the study (or into the analysis).
D4: Bias due to post-exposure interventions.
D5: Bias due to missing data.
D6: Bias arising from measurement of the outcome.
D7: Bias in selection of the reported result.

Judgement
! Very high
X High
- Some concerns
+ Low

© 2025 Wennberg E et al. *JAMA Network Open*.

From: McGuinness, LA, Higgins, JPT. Risk-of-bias VISualization (robvis): An R package and Shiny web app for visualizing risk-of-bias assessments. Res Syn Meth. 2020; 1- 7. https://doi.org/10.1002/jrsm.1411

Overall risk of bias ratings were based on the highest risk of bias rating in any individual domain, with the exception of studies whose highest risk of bias rating was present across ≥3 domains; these were given overall ratings at the next highest risk of bias.

© 2025 Wennberg E et al. *JAMA Network Open*.

eFigure 2. Random effects meta-analysis of included studies of adult patients facing and not facing language barriers that reported adjusted odds ratios of non-completion vs completion of scheduled virtual care visits

A. Studies of primary and/or specialist care (n=3).

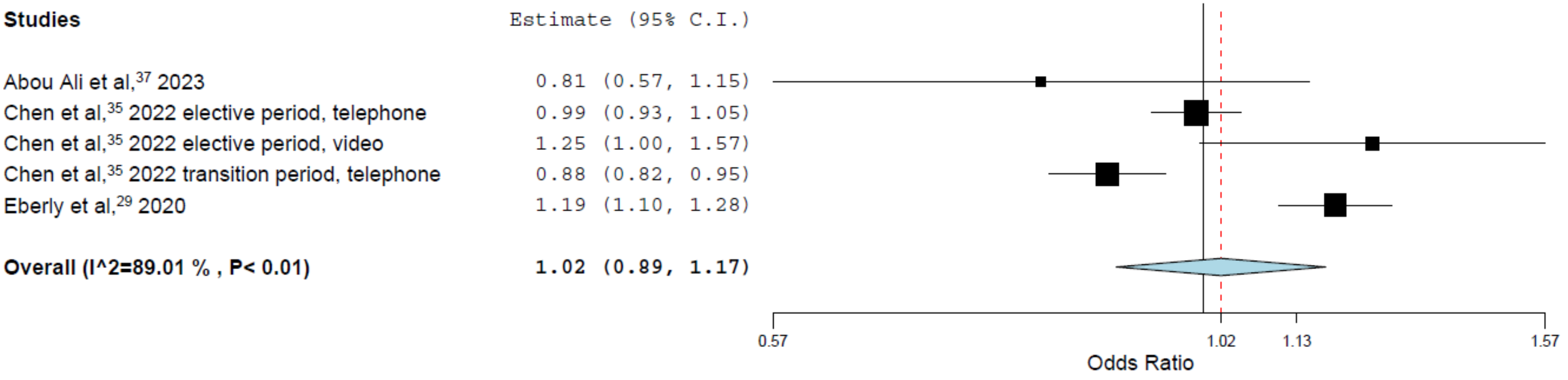

B. Studies of primary care (n=2).

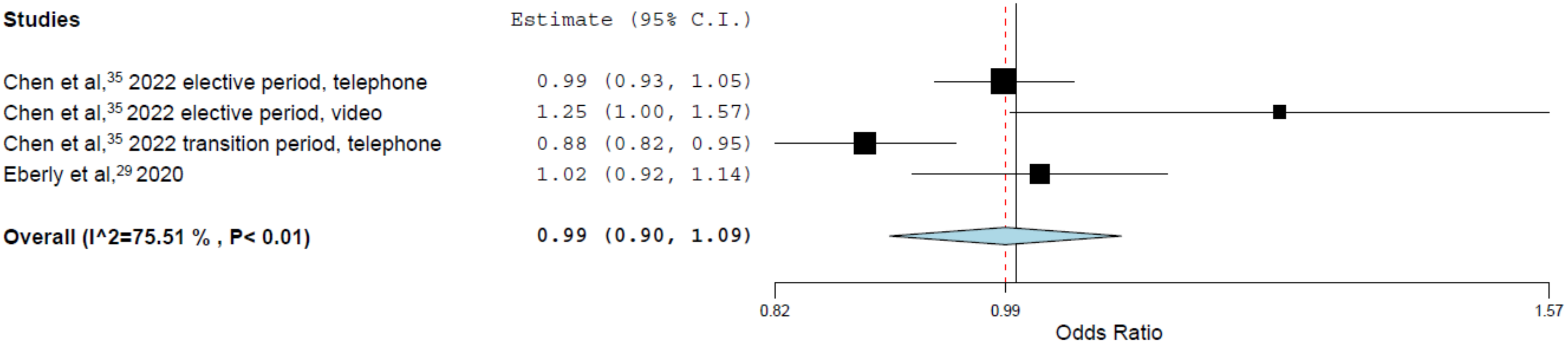

© 2025 Wennberg E et al. *JAMA Network Open*.

C. Studies of specialist care (n=2).

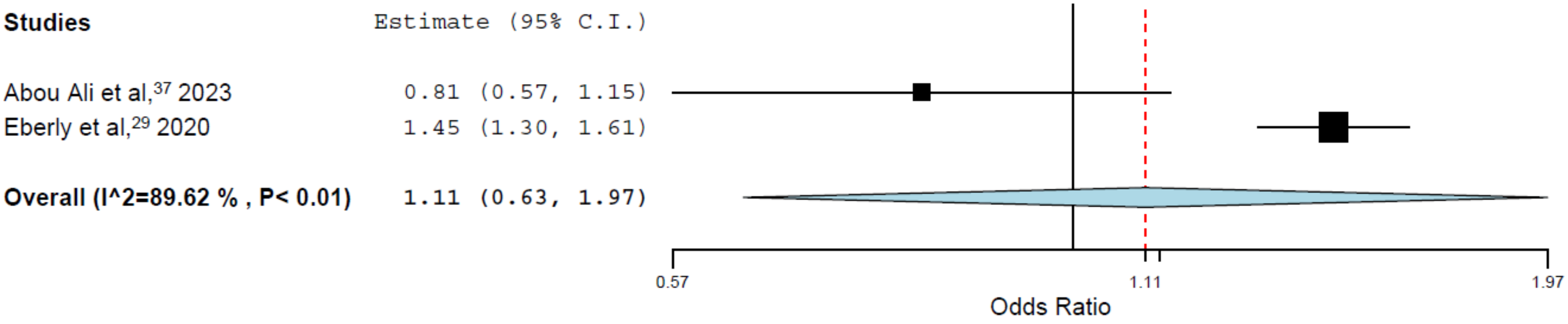

Chen 2022 included three independent subgroups; these were treated as separate studies.

© 2025 Wennberg E et al. *JAMA Network Open*.

eFigure 3. Random effects meta-analysis of included studies of adult patients facing and not facing language barriers that reported unadjusted odds of non-completion vs completion of scheduled virtual care visits

A. Studies of primary and/or specialist care (n=5).

Studies	Estimate (95% C.I.)	Ev/Trt	Ev/Ctrl
Bell et al,[38] 2022	0.78 (0.47, 1.28)	34/130	60/192
Eberly et al,[29] 2020	1.26 (1.18, 1.34)	1990/3895	65280/144099
Kim et al,[26] 2022b	1.02 (0.53, 1.96)	19/130	24/167
Pitaro et al,[36] 2022	0.34 (0.21, 0.55)	22/153	370/1122
Gmunder et al,[34] 2021	1.25 (1.23, 1.27)	41082/101950	91191/259714
**Overall (I^2=87.99 % , P< 0.01)**	**1.07 (0.91, 1.25)**	**43147/106258**	**156925/405294**

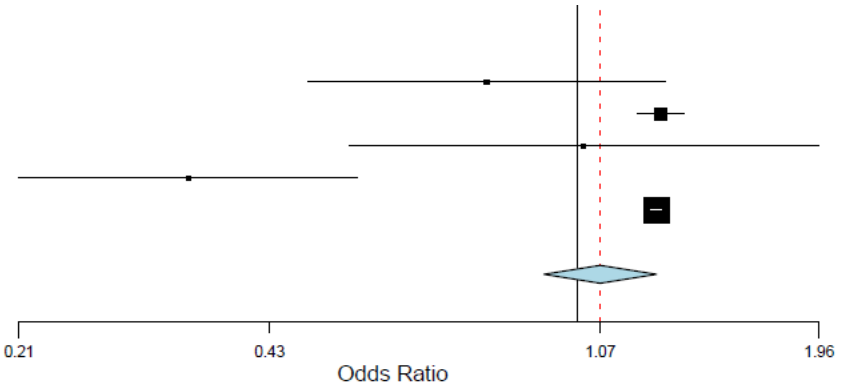

B. Studies of specialist care (n=4).

Studies	Estimate (95% C.I.)	Ev/Trt	Ev/Ctrl
Bell et al,[38] 2022	0.78 (0.47, 1.28)	34/130	60/192
Eberly et al,[29] 2020	1.64 (1.49, 1.80)	1087/1819	33413/70334
Kim et al,[26] 2022b	1.02 (0.53, 1.96)	19/130	24/167
Pitaro et al,[36] 2022	0.34 (0.21, 0.55)	22/153	370/1122
**Overall (I^2=93.98 % , P< 0.01)**	**0.83 (0.38, 1.82)**	**1162/2232**	**33867/71815**

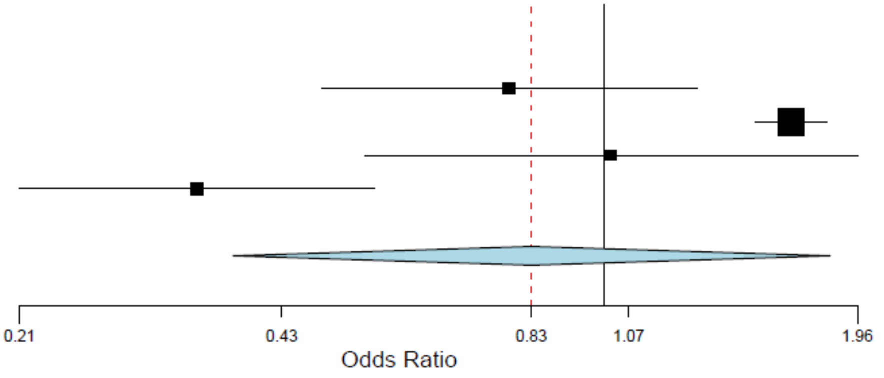

© 2025 Wennberg E et al. *JAMA Network Open*.

eFigure 4. Random effects meta-analysis of included studies (n=2) of adult patients facing and not facing language barriers that reported adjusted odds ratios of ever versus never use of virtual primary and specialist care

Studies	Estimate (95% C.I.)
Chang et al,[40] 2022	0.86 (0.64, 1.15)
El−Toukhy et al,[41] 2020	0.80 (0.49, 1.30)
**Overall (I^2=0 % , P=0.80)**	**0.84 (0.66, 1.08)**

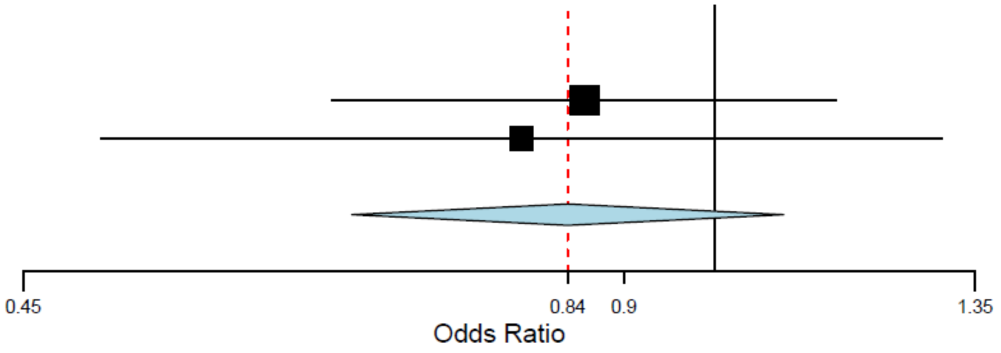

© 2025 Wennberg E et al. *JAMA Network Open*.

eFigure 5. Random effects meta-analysis of included studies (n=4) of adult patients facing and not facing language barriers that reported unadjusted odds of ever versus never use of virtual primary and/or specialist care

Studies	Estimate (95% C.I.)	Ev/Trt	Ev/Ctrl
Andersen et al,[39] 2021	3.99 (1.13, 14.08)	5/13	13/96
Le et al,[30] 2022	1.51 (1.44, 1.57)	3481/11451	17235/76746
Ramsey et al,[43] 2022	1.09 (0.87, 1.37)	100/440	1639/7707
Rodriguez et al,[42] 2021	0.34 (0.31, 0.38)	366/8063	9381/76356
**Overall (I^2=99.53 % , P< 0.01)**	**1.11 (0.42, 2.94)**	**3952/19967**	**28268/160905**

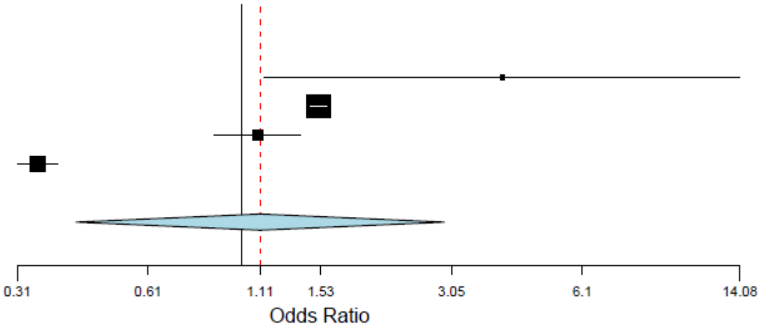

© 2025 Wennberg E et al. *JAMA Network Open*.

eFigure 6. Random effects meta-analysis of included studies (n=3) of caregivers of pediatric patients facing and not facing language barriers that reported unadjusted odds of use of virtual versus in-person specialist care

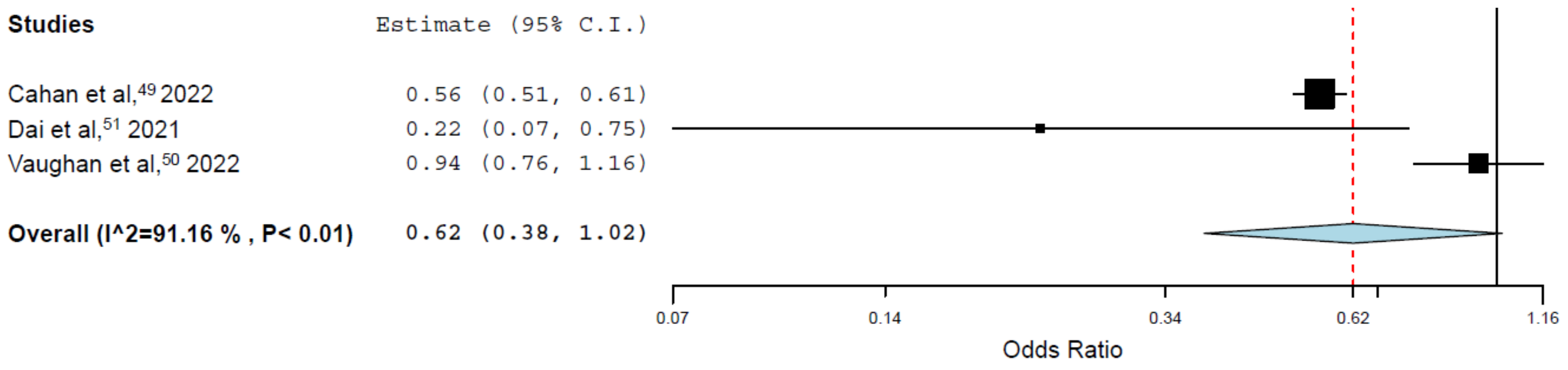

© 2025 Wennberg E et al. *JAMA Network Open*.
