# Supplementary material for: Virtual Care Among Adults Facing Language Barriers: A Systematic Review and Meta-Analysis
Source: JAMA Netw Open. 2025 Jun 5;8(6):e2513906. doi: 10.1001/jamanetworkopen.2025.13906 (PMC12142448; doi:10.1001/jamanetworkopen.2025.13906)
Supplement: Supplement 2. — Data Sharing Statement [file jamanetwopen-e2513906-s002.pdf]

Data Sharing Statement

Wennberg. Virtual Care Among Adults Facing Language Barriers. *JAMA Netw Open*. Published June 05, 2025. doi:10.1001/jamanetworkopen.2025.13906

Data
Data available: Yes
Data types: Other (please specify)
Additional Information: Data extraction and quality assessment forms, search strategies
How to access data: erica.wennberg@mail.utoronto.ca Search strategies available in the supplement.
When available: With publication

Supporting Documents
Document types: None

Additional Information
Who can access the data: anyone requesting
Types of analyses: N/A
Mechanisms of data availability: Search strategies are available in the supplement. Data extraction and quality assessment forms will be made available at request.
